# Transcriptomic and Genomic Testing to Guide Individualized Treatment in Chemoresistant Gastric Cancer Case

**DOI:** 10.3390/biomedicines8030067

**Published:** 2020-03-23

**Authors:** Alexey Moisseev, Eugene Albert, Dan Lubarsky, David Schroeder, Jeffrey Clark

**Affiliations:** 1 Institute for personalized medicine, I.M. Sechenov First Moscow State Medical University, 119048 Moscow, Russia; mormegill@mail.ru; 2 Quantida, Inc., Newton, MA 02466, USA; dan@difive.com; 3 Wellesley Internal Medicine, 372 Washington St Ste 2, Wellesley Hills, MA 02481, USA; ds@wintmed.com; 4 Department of Hematology and Oncology, Massachusetts General Hospital, 55 Fruit Street Boston, MA 02114, USA; Clark.Jeffrey@mgh.harvard.edu

**Keywords:** gastric cancer, stomach adenocarcinoma, genomic test, molecular pathway analysis, immunotherapy, platform comparison, mutation, expression analysis, companion diagnostics

## Abstract

Gastric cancer is globally the fifth leading cause of cancer death. We present a case report describing the unique genomic characteristics of an Epstein–Barr virus-negative gastric cancer with esophageal invasion and regional lymph node metastasis. Genomic tests were performed first with the stomach biopsy using platforms FoundationOne, OncoDNA, and Oncopanel at Dana Farber Institute. Following neoadjuvant chemotherapy, residual tumor was resected and the stomach and esophageal residual tumor samples were compared with the initial biopsy by whole exome sequencing and molecular pathway analysis platform Oncobox. Copy number variation profiling perfectly matched the whole exome sequencing results. A moderate agreement was seen between the diagnostic platforms in finding mutations in the initial biopsy. Final data indicate somatic activating mutation Q546K in *PIK3CA* gene, somatic frameshifts in *PIH1D1* and *FBXW7* genes, stop-gain in *TP53BP1*, and a few somatic mutations of unknown significance. RNA sequencing analysis revealed upregulated expressions of *MMP7, MMP9, BIRC5,* and *PD-L1* genes and strongly differential regulation of several molecular pathways linked with the mutations identified. According to test results, the patient received immunotherapy with anti-PD1 therapy and is now free of disease for 2 years. Our data suggest that matched tumor and normal tissue analyses have a considerable advantage over tumor biopsy-only genomic tests in stomach cancer.

## 1. Introduction

Gastric cancer arises from epithelial lining of the stomach and is globally the fifth leading cause of cancer and the third leading cause of cancer death, making up 7% of cases and 9% of deaths [1]. Major classifications of gastric cancers (GCs) include histological classification by Lauren [2], where GCs are subdivided into intestinal and diffuse histotypes, and molecular classification recently proposed by The Cancer Genome Atlas (TCGA) consortium, which includes chromosomal instability, microsatellite instability (MSI), genomically stable, and Epstein–Barr virus (EBV) positive GC types [3]. EBV-positive subtype covers ~9%–10% of all SC cases and is ~two times more frequent in male than in female patients. Diffuse and intestinal histotypes are presented in EBV-positive GCs in equal proportions [4]. In 30%–40% of the cases, this subtype overexpresses PD-L1 (ligand of Programmed Death-1) protein [5]. Enhanced expression of this biomarker may be associated with poor survival [6]. However, targeted PD-L1-specific immunotherapy showed 22% objective response, 24% progression-free survival, and 69% overall survival 6 months after treatment [7].

The relatively high mortality in GC is linked with its strong potential to form metastases, mainly via epithelial–mesenchymal transition (EMT) process when epithelial cells lose their polarity and adhesion features while gaining migratory and invasive properties to become mesenchymal stem-like cells [8].

Prognosis in locally advanced and metastatic gastric cancer is dismal, with 1-year and 2-year progression-free survival of barely 50% and 25%, respectively, and 5-year overall survival less than 10% [9]. Standard treatment for operable tumors consists of surgery with perioperative chemotherapy, the impact of radiotherapy being controversial. Recently, much effort was made to improve these dismal results with immune checkpoint inhibitors, mostly anti-PD-1 agents such as Pembrolizumab and Nivolumab. Several markers were explored to predict tumor response to these drugs, including, among others, tumor mutation burden and immunohistochemical tests for PD-L1. Unfortunately, apart from MSI, robust response predictors are lacking, and the optimal ways to incorporate immunotherapy into treatment algorithm are still under investigation [10,11,12].

We present a case of locally advanced gastric cancer treated with sequential chemotherapy, surgery, and immunotherapy; those prescriptions were guided by extensive genomic and transcriptomic testing. The genetic tests used were based on the gene panel exome sequencing [13,14], whole exome sequencing [15,16], and a combination of RNA sequencing and whole exome sequencing [17,18], including molecular pathway annotation algorithms [19,20,21]. This allowed to compare different methods of annotating tumor-specific mutations and to implement gene expression-based personalized prioritizing of targeted therapeutics.

To our knowledge, this is the first report simultaneously using alternative comprehensive genomic, copy number variation, and transcriptomic approaches to characterize primary and metastatic disease in both tumor-only and matched tumor-normal modes to identify putative treatment options for the individual patient with GC.

## 2. Case Presentation

In October 2017, an 80-year-old Caucasian woman presented with gastric tumor that was incidentally discovered during routine ultrasonography. Biopsy showed moderately to poorly differentiated adenocarcinoma and HER2- and MSI-negative. Tests for *H. pylori* and EBV were negative. Staging with endoscopy, endoscopic ultrasonography, magnetic resonance imaging (MRI), and positron emission tomography–computed tomography (PET-CT) with 18F-fluoro-2-deoxy-d-glucose (^18^FDG) revealed esophageal extension and suspicious regional lymph nodes, corresponding to clinical stage T3N1M0. The council decision was to start induction chemotherapy.

**Figure 1:**
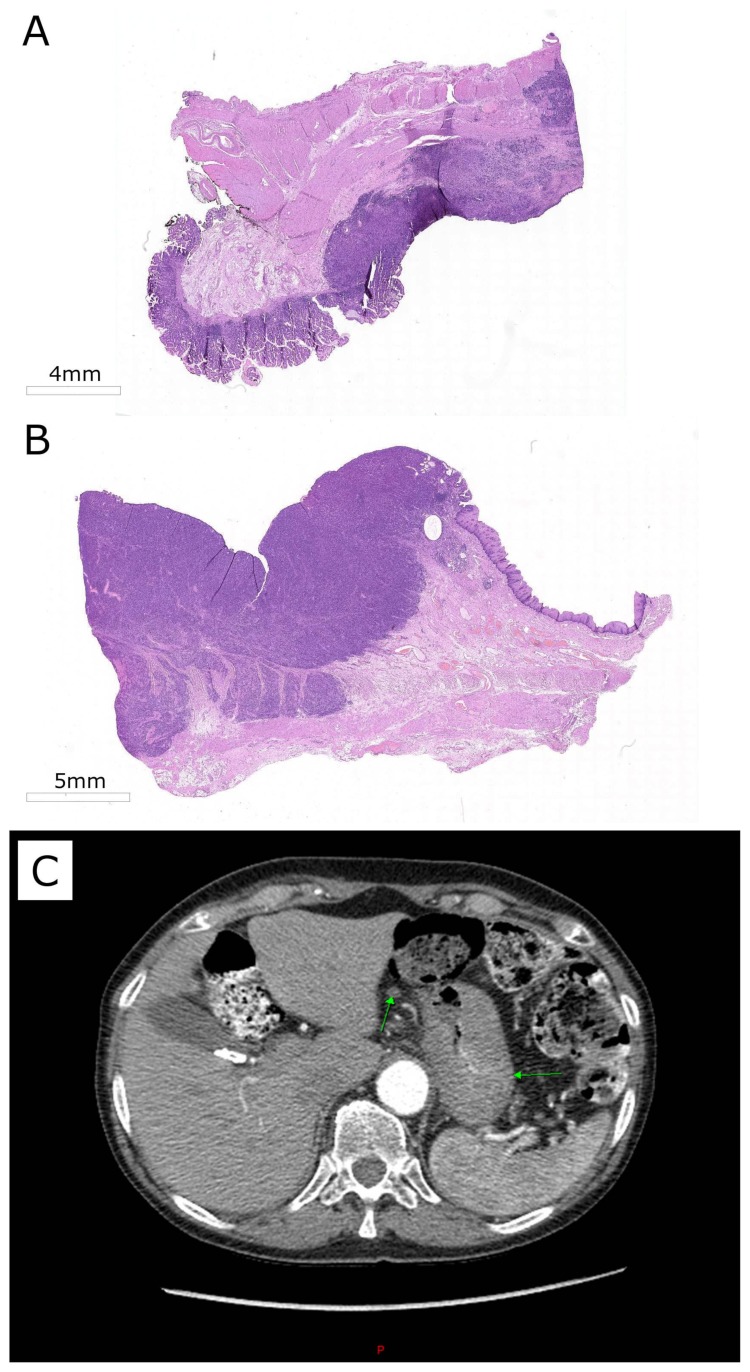
(**A**,**B**) Hematoxylin and eosin (H&E) staining shows moderately to poorly differentiated adenocarcinoma. (**A**) Tumor removed from stomach. (**B**) Tumor removed from esophagus. (**C**) Baseline tumor appearance: primary gastric lesion (right arrow) and involved lymph node (left arrow).

In November 2017, two courses of the FOLFOX regimen (Oxaliplatin + Fluorouracil/Leucovorin) were administered. The treatment was tolerated well, and the regimen was escalated to FLOT (Docetaxel, Oxaliplatin, and Fluorouracil/Leucovorin) by adding docetaxel; 4 cycles were given till February 2018. Toxicity was moderate, mainly neutropenia managed with filgrastim. Endoscopy after 4 cycles showed tumor shrinkage from 6 to 4 cm but no further regression after 6 cycles. In March 2018, partial proximal gastrectomy with esophageal resection and lymph node dissection was performed. Pathology reported viable residual tumor and one involved node, ypT3N1 (Figure 1).

According to current guidelines, further management would be adjuvant chemotherapy (4–6 cycles of FLOT or another regimen), but the patient’s age and lack of clear benefit (downstaging) from the previous cytostatic treatment prompted us to seek alternative approaches. Another option could be observation until disease relapse, but the patient pursued an active treatment strategy. While she had a positive immunohistochemistry for PD-L1, this result was of limited practical value because this marker performs poorly in GC; moreover, at the time of treatment, immune checkpoint inhibitors like Pembrolizumab were not sufficiently studied and not approved for adjuvant therapy in gastric cancer. Along with PD-L1 positivity, patient’s age and moderate mutation burden suggested benefit from immunotherapy, but the evidence was inconclusive [10,11,12].

To guide further therapy, the patient consented to enter a clinical trial NCT03724097 and underwent testing with Oncobox platform. Oncobox algorithm builds a personalized rating of target drugs for individual cancer patients. It is based on a simultaneous analysis of gene expression and molecular pathway activation in the patient’s tumor and was shown to be effective in a retrospective cohort of gastric cancer patients [22], in several prospective advanced cancers cases [23,24,25], and in an ongoing prospective clinical investigation [26]. Gene expression profiling and whole exome sequencing was approved by institutional Review Board (IRB) at Clinical Center Vitamed, Moscow, Russia, protocol date 17 October 2016. According to test results (see below in details), further chemotherapy was omitted, and instead of it, adjuvant immunotherapy with anti-PD-1 antibody Pembrolizumab (Keytruda™) was prescribed. From June to December, the patient received 8 cycles. Adverse events were limited to hypothyroidism and mild fatigue. As of February 2020, she is free of disease with Karnofsky index 80%.

### 2.1. High Throughput Molecular Characterization of Tumor Biosamples

Genomic tests were performed first with the stomach biopsy using commercial platforms Foundation One (F1) and OncoDNA, and copy number variation (CNV) was assessed using Oncopanel at Dana Farber Institute (Supplementary File 1) [19,27,28,29,30,31,32,33,34,35,36,37,38,39,40,41,42,43,44,45,46,47]. Following neoadjuvant chemotherapy, residual tumor was resected; the stomach and esophageal residual tumor samples were obtained and compared with the initial biopsy by whole exome sequencing and RNA sequencing-based molecular pathway analysis platform Oncobox (Obx). This allowed to identify the most probable individual case driver mutations using four alternative methods, to measure tumor mutational burden (TMB) using two methods, to establish gene expression levels of tumor marker genes, and to assess potential individual utility of immunotherapy using molecular pathway analysis.

### 2.2. Tumor-Only and Tumor-Normal Modes for Finding Mutations in Tumor Biopsy and Surgery Biosamples

WES-Obx pipeline was then applied to identify mutations using additional biosamples obtained from the same patient during surgical removal of the tumor. Two new tumor biosamples were obtained for stomach and esophageal localizations, respectively, matched by adjacent normal tissue samples. Totally, this made three tumor samples (initial biopsy, surgical stomach, and surgical esophageal) and two normal tissue samples (from stomach and esophageal tissues). Whole exome sequencing (WES) was done for all those biosamples (Table 1), and WES-Obx platform was used to identify mutations. Whole exome sequencing and gene expression data were deposited to NCBI Sequence Read Archive (SRA) under the accession numbers PRJNA545281.

In the previous tests comparing results of different platforms for the same tumor biopsy sample, we were able to identify mutations but could not find out if they were of germline or somatic origin. Now, we had reference normal patient’s tissues and could distinguish somatic mutations. WES-Obx platform was applied in tumor-only mode for the sole tumor biopsy sample, thus giving an output of 502 germline + somatic mutations (Supplementary File 2), and in the mode of comparison with reference normal tissues (tumor-reference mode), thus providing 37 and 38 *somatic* mutations identified for surgical tumor samples of esophageal and stomach localizations, respectively. This dramatic difference in the number of mutations identified most probably pointed to the extremely high proportion (>90%) of germline mutations and/or formalin fixation artifacts in the tumor-only mode of analysis. Most of mutations identified in tumor-reference mode were also identified in a tumor-only mode. Differences between somatic mutations found for the esophageal and stomach tumor localizations may be linked with different cellular compositions of those samples and overall patient’s tumor heterogeneity. We, therefore, concluded that, in this case, tumor-reference mode of mutation search was more informative than a tumor-only mode. Final data indicate somatic activating mutation Q546K in the *PIK3CA* gene, somatic frameshifts in the *PIH1D1* and *FBXW7* genes, stop-gain in *TP53BP1*, and a few somatic mutations of unknown significance (Supplementary Files 1, 3 and 5).

### 2.3. Tumor Mutational Burden Analysis

Tumor mutational burden (TMB) is an emerging cancer biomarker that can influence therapeutic strategy, particularly in selection of immunotherapeutic treatment options [48]. In the F1 report, for the patient’s primary tumor biopsy, TMB measured was 6 mutations per million nucleotide bases (per mb). In WES-Obx analysis for the same biopsy sample, a similar figure was obtained of 7.8 mutations per mb. However, for the surgical tissue material, lower TMB values were obtained using WES-Obx platform: 5.7 and 5.0 for esophageal and stomach segments of tumor, respectively. These lower values were in a good agreement with extremely high degrees of surgical tumor tissue infiltration by blood cells (Figure 1), where only ~50% were tumor cells according to histopathological expertise. Low proportion of tumor cells hampers robust identification of mutations due to low coverage of mutant alleles. However, this was not the case for the primary tumor biopsy, and we therefore used the TMB value of 6–7.8 as the reference for the patient’s tumor case.

### 2.4. Analysis of Molecular Pathway Activation

**Figure 2:**
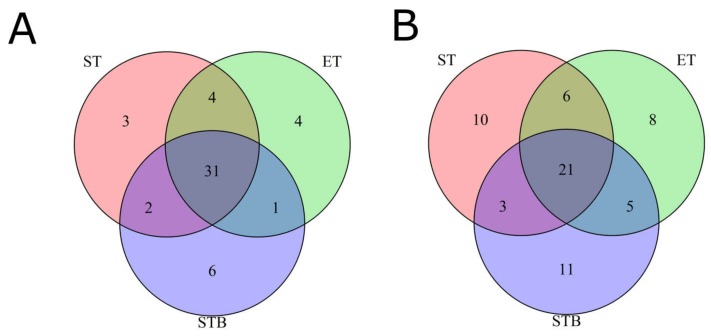
Overlap of top 40 activated or downregulated pathways for Stomach tumor (ST), Esophagus tumor (ET), and Stomach tumor biopsy (STB) samples: (**A**) upregulated pathways and (**B**) downregulated pathways.

We examined the expression of genes, previously reported to be upregulated in gastric cancer. We identified significantly upregulated expression of *MMP7, MMP9, BIRC5*, and *PD-L1*. To investigate functional interplay of the discovered driver gene mutations with the gene expression profiles, we analyzed differentially regulated molecular pathways using Oncobox pathway analysis software [23,24]. For every pathway, a *pathway activation level* (PAL) value was calculated [49] corresponding to degree of a pathway activation or downregulation in a tumor sample (biopsy, stomach, and esophageal localizations) in comparison to the normal stomach sample. Overall, PAL signatures strongly correlated among the three tumor samples analyzed (pairwise Spearman correlation coefficients 0.87–0.89). We then compared top 40 most strongly up- and downregulated molecular pathways in different tumor biosamples. Triple intersection showed that, among them, there were top common 31 upregulated and 21 downregulated pathways (Figure 2; Supplementary File 4). 

**Figure 3:**
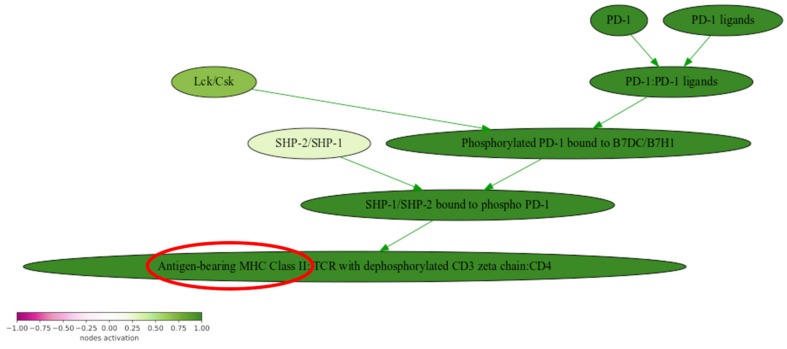
Pathway activation profile of the “Reactome PD-1 signaling Main Pathway” in a stomach tumor sample: mutated genes are circled in red.

Noteworthily, the second top upregulated molecular pathway was clinically actionable “Reactome PD1 signaling Pathway”, which also contained mutated gene *HLA-DRB1* (Figure 3). Upregulation of this pathway along with apparently high tumor infiltration by blood indicated abnormal activation of PD1/PD-L1 immunosuppression mechanisms and suggested potential benefit for the respective targeted immunotherapeutic treatment.

## 3. Discussion 

We compared whole exome sequencing profiles of tumor biopsy and two surgical tissue samples from different tumor sites (stomach and esophagus) in comparison with two adjacent normal tissue samples. We also compared performances of three commercial genetic diagnostics panels: FoundationOne, OncoDNA, and WES-Oncobox. Totally, we identified 502 mutations (Supplementary File 2) and found 3 major candidate genes—*FBXW7*, *PIH1D1*, and *TP53BP1*—that may serve as tumor suppressors and may contain inactivating mutations (Supplementary File 1). We found that these genes underwent copy-neutral loss of heterozygosity, which points to driver function of these mutations.

We detected significantly upregulated expression of *MMP7, MMP9, BIRC5*, and *PD-L1* in the patient’s cancer samples. Increased expression of *PD-L1* was also associated with strong upregulation of the PD1 signaling molecular pathway, which supported the idea that immunotherapy could potentially be effective in this case. Taken together with intermediate tumor mutation burden value of ~7 mutations per mb, these results supported including anti-PD1/PD-L1 therapy in the patient’s treatment plan. Normal tissue was required for the pathway analysis, as Oncobox bioinformatical platform calculates pathway activation level in a tumor sample compared to a normal sample.

The genetic tests and advanced data analytic tools used here are still far less expensive than the therapeutics and/or clinical procedures applied. They also have a strong potential to decrease overall costs associated with the treatment by timely selecting the most appropriate therapeutic strategy. The prices for the genetic tests employed varied ~$2000–6000 per test with the complete Oncobox test including drugs prioritization based on the combination of whole-exome sequencing and RNA sequencing data amounting to $3500 [50]. 

## 4. Conclusions

In conclusion, the presented case report suggests that matched tumor and normal tissue analyses may have considerable advantage over biopsy-only genomic tests in selected cases. However, further prospective clinical investigations involving larger groups of patients with different malignancies are required to estimate clinical utility of such approach.

## Figures and Tables

**Table 1:** Statistics of genomic data obtained using sequencing-based cancer diagnostics platform Oncobox.

	Biopsy	Normal Stomach	Tumor—Stomach	Normal Esophagus	Tumor—Esophageal
Number of reads	222 × 10^6^	227 × 10^6^	236 × 10^6^	139 × 10^6^	232 × 10^6^
Reads lengths	30–150	30–150	30–150	30–150	30–150
Reads mapped on exons of protein coding genes (%)	62	35	37	40	40
Mean per-base coverage in exons of protein coding genes (standard deviation)	147 (283)	112 (211)	99 (196)	65 (155)	108 (244)
Reads in detected genes (%)	87	69	70	70	72

## Supplementary Materials

The following are available online at https://www.mdpi.com/2227-9059/8/3/67/s1, Supplementary File 1: Technical comparison of DNA sequencing-based diagnostic platforms WES-Oncobox, OncoDNA, and FoundationOne; Supplementary File 2: Output of WES-Obx platform in tumor-only mode for the sole tumor biopsy sample: 502 germline + somatic mutations; Supplementary File 3: 386 mutations, common for all analyzed tumor samples. Supplementary File 4: Top 40 most strongly up- and downregulated molecular pathways. Supplementary File 5: 137 “off-target” genes in F1 sequencing results.
