# Supplementary material for: Transcriptomic and Genomic Testing to Guide Individualized Treatment in Chemoresistant Gastric Cancer Case
Source: Biomedicines. 2020 Mar 23;8(3):67. doi: 10.3390/biomedicines8030067 (PMC7148467; doi:10.3390/biomedicines8030067)
Supplement: Supplementary file 1 [file biomedicines-08-00067-s001.zip › biomedicines-742411-supplementary/Supplementary file 1.docx]

**Supplementary file 1. Technical comparison of DNA sequencing-based diagnostic platforms WES-Oncobox, OncoDNA and FoundationOne**

1. **Exome sequencing of tumor biopsy sample**

To identify substitutions, insertion and deletion mutations in tumor biopsy sample, we used two commercial platforms: OncoDNA (OD), FoundationOne (F1) for target gene panels, did whole exome sequencing (WES) and analyzed data using Oncobox (WES-Obx) platform. Specifically, OD did exon sequencing for a panel of 75 genes, F1- 315 genes plus analyzed rearrangements in 28 genes, and more that 22.500 genes were investigated in a WES-Obx analysis. Primary sequencing data were obtained for every assay and then compared using uniform sequencing alignment and mapping algorithm (Table S1).

For direct comparison of sequencing data, we took 73 genes that were sequenced simultaneously by all three platforms.

*Table S1. Statistics of genomic data obtained using sequencing-based cancer diagnostics platforms.*

|  | **Oncobox** | **OncoDNA** | **FoundationOne** |
| --- | --- | --- | --- |
| Number of genes covered | 22 500 | 75 | 315 (+rearrangements) |
| Total number of sequencing reads | 222*10^6^ | 7,5*10^6^ | 90*10^6^ |
| Sequencing reads lengths | 30-150 | 25-353 | 49 |
|  |  |  |  |
| Reads mapped on exons of protein coding genes | 62% | 85% | 31% |
| Mean per-base coverage in exons of protein coding genes (standard deviation) | 127 (256) | 1046 (5884) | 457 (561) |
|  |  |  |  |
| Reads in human genes | 88% | 87% | 47% |

**Analysis of mutations detected using different platforms**

In the reports received, WES-Obx analysis detected 502 mutations, F1 – sixteen and OD – only two (Supplementary file 2). In the common genes included in all three panels, there were four mutations found in the WES-Obx and F1 reports, but at the same time only two in the OD report. Manual analysis of raw sequencing data showed that this shortage was due to lack of several gene exons in the OD sequencing panel, including those where mutations were detected using alternative platforms (Figure S1). Since OD platform results were the least informative, we next focused on the comparison of WES-Obx and F1 reports for the set of common genes.


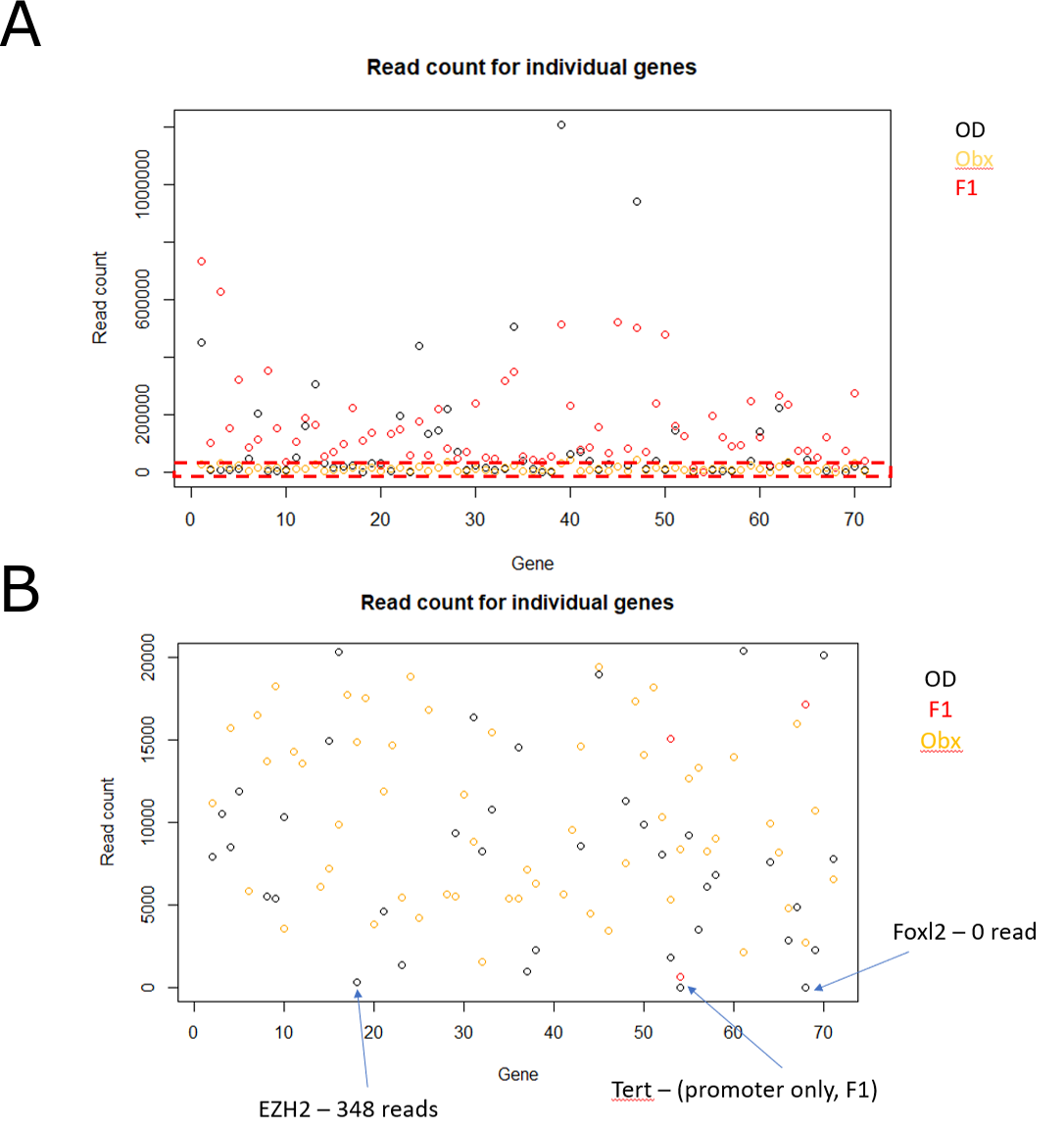


*Figure S1. Number of reads per gene. A – all genes, B – magnified view from A.* The data were generated using bedtools coverage software.

Totally, 22 mutations were identified, of them 17 in WES-Obx and 16 in F1 platform reports (Table S2). The mutations detected were manually analyzed using raw sequencing reads obtained by each platform and a list of 16 mutations confirmed by sequencing data from both platforms was generated (Table S2, positions 1-16). The remaining six mutations could not be confirmed by data from the alternative platform. The percentage of errors was 5/17 (~29%) for WES-Obx and 6/16 (~38%) for F1 platforms (Table S2). The other few hundred mutations identified in WES-Obx assay (Supplementary file 2) could not be independently investigated using an alternative platform.

*Table S2. Mutations independently investigated using WES-Obx and F1 platforms.*

| **No** | **Gene/mutation ID** | **Presence in WES-Obx report** | **Presence in F1 report** | **Manual inspection of WES-Obx and F1 raw sequencing data, result** |
| --- | --- | --- | --- | --- |
| *1* | *MLL2, G1234A* | + | + | + |
| *2* | *DNMT3A, W297S* | + | + | + |
| *3* | *PIK3R2, S276N* | + | + | + |
| *4* | *FANCA, R1053C* | + | + | + |
| *5* | *RET, E843K* | + | + | + |
| *6* | *SPTA1, R60** | + | + | + |
| *7* | *PTPN11, D61Y* | + | + | + |
| *8* | *PIK3CA, Q546K* | + | + | + |
| *9* | *FBXW7, D126FS*4* | + | + | + |
| *10* | *FOXP1, N570S* | + | + | + |
| *11* | *TSC1, K587R* | + | + | + |
| *12* | *BARD1, C557S* | + | - | + |
| *13* | *CTNNA1, A179V* | + | - | + |
| *14* | *SUFU, A340S* | + | - | + |
| *15* | *ABL1, E3A* | - | + | + |
| *16* | *EPHA5, V647_R648>IL* | - | + | + |
| *17* | *CBL,E366fs* | + | - | - |
| *18* | *DDR2, A747T* | + | - | - |
| *19* | *FGFR2, T763fs* | + | - | - |
| *20* | *ZNF217, D323N* | - | + | - |
| *21* | *AKT3, putative splice site 562-1G>A* | - | + | - |
| *22* | *IRF4, D33N* | - | + | - |

1. **Quality check for WES-Oncobox (Obx), OncoDNA (OD) and FoundationOne (F1) raw sequencing data**

For OD and F1, preprocessed bam files were used as the input. The analysis performed by Fastqc program demonstrated high quality of all analyzed raw data files (Figure S2). Results obtained for OD markedly differed from the other two platforms due to the different sequencing technologies used for OD (Ion Torrent) vs Obx and F1 (Illumina HiSeq). Quality results from OD were typical for Ion Torrent sequencing method, e.g. [PMID 26057250] and could be partially explained by the fact that historically Fastqc software was designed for the analysis of raw data generated by Illumina sequencing engines. We concluded, therefore, that the initial data quality for all three platforms met the commonly accepted quality standards and were sufficient for further analyses.


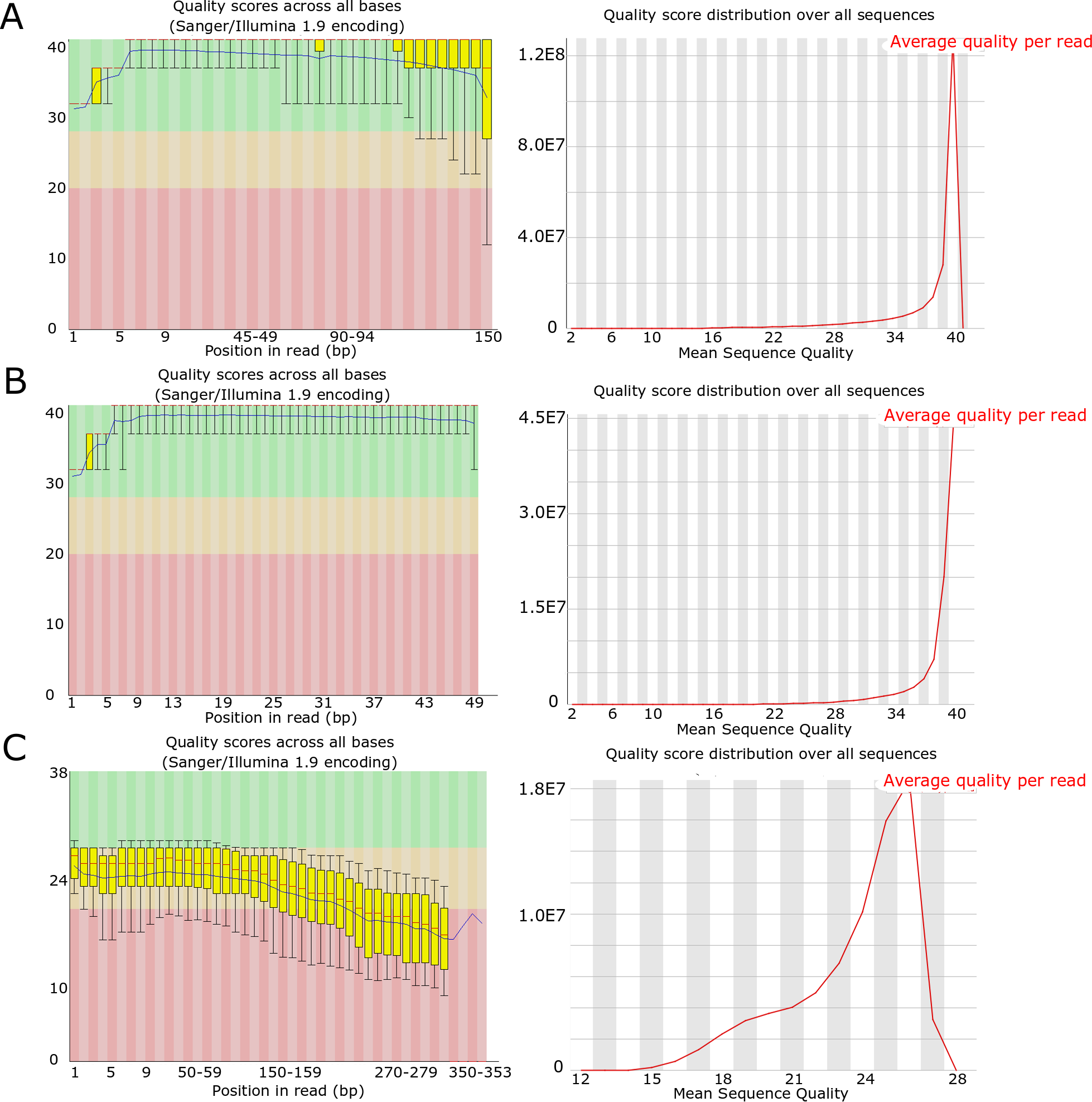


*Figure S2. Per-base/per-sequence quality scores observed for the obx(A), F1 (B) and OD (C) sequencing data.*

1. **Coverage of targeted genes**

Average coverages for targeted genes (all known human protein coding genes in case of WES-Obx, 75 for OD and 315 for F1) were roughly comparable among all three platforms. WES-Obx had the lowest coverage and OD had a highest mean coverage but also an outstandingly high standard deviation. For direct comparison of exons coverages, we took 73 genes available simultaneously on all three sequencing platforms tested here. A substantial difference was seen in coverage distributions across the exons (Figure S3). Obx and F1 sufficiently covered ~ 50-60% of all gene exons whereas for OD this proportion was less than 20%. On the other hand, in case of OD the highest per-base coverage depth was obtained (Figure S4).

*
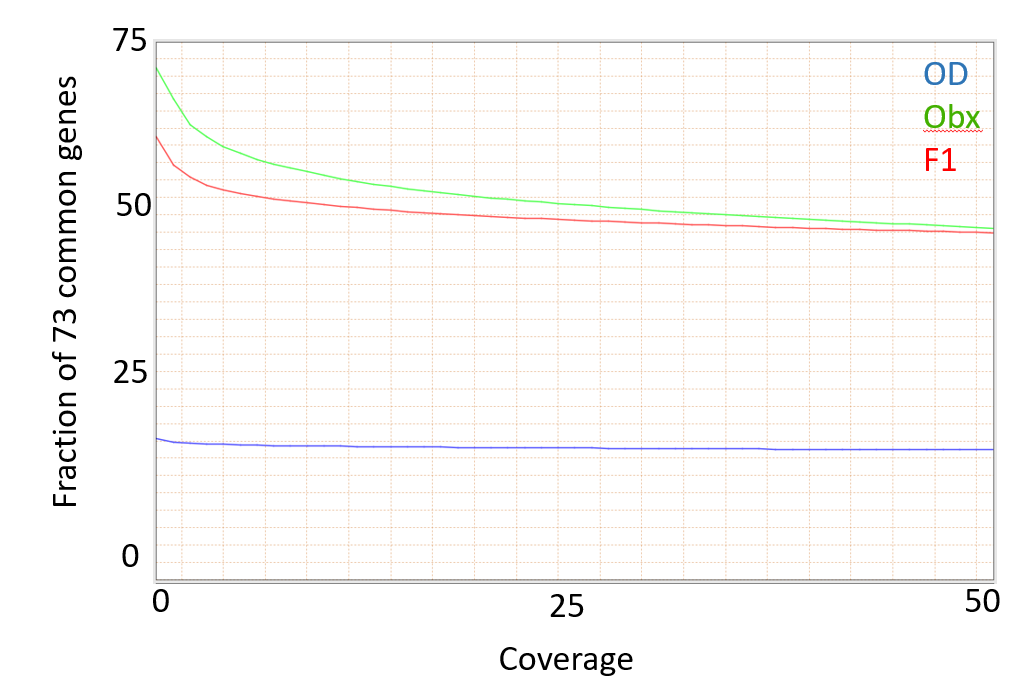
*

*Figure S3. Exon coverage for 73 common genes for WES-Obx, OD and F1 platforms. The data were visualized using qualimap software.*

*
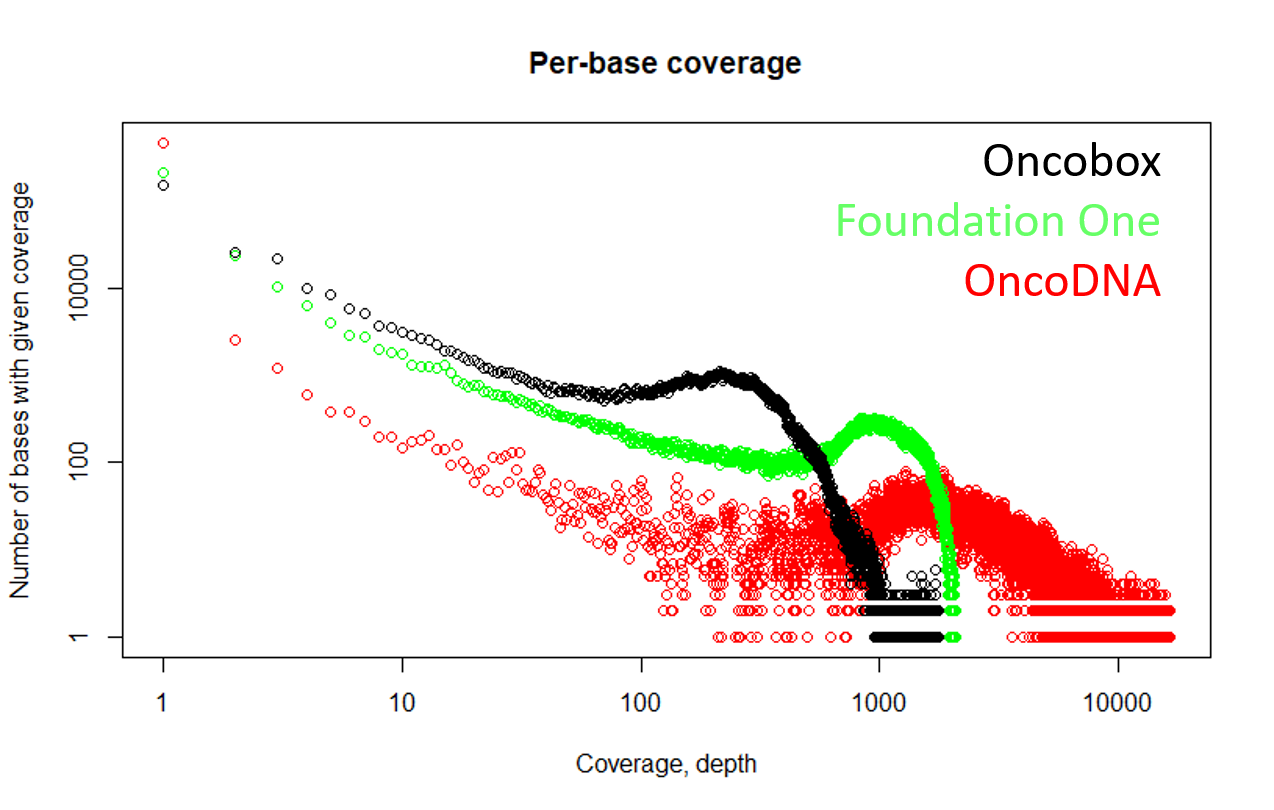
*

*Figure S4. Distribution of per-base sequencing coverages for exons of 73 common genes for WES-Obx, OD and F1 platforms. Data were generated by qualimap software and visualized using R programming language.*

Averaged coverages for the individual genes obtained using these platforms are shown on Figure S1. The lowest dispersion was seen for WES-Obx (2*10^3^ - 20*10^3^ reads per gene), whereas for the other platforms it varied more significantly: 0-10^6^ (OD) and 15*10^3^ - 5*10^5^ (F1). The highest median coverage was detected for F1 platform. Noteworthy, different platforms also covered different numbers of target gene exons with the highest proportion for WES-Obx and the lowest for OncoDNA platform (Figure S5).

Surprisingly, for the OD platform, the sequence quality analysis revealed that exons for two genes listed as “reported” (*TERT* and *FOXL2*) were not covered at all (had zero coverage in OD raw data). F1 platform also had outstandingly low coverage for *TERT* gene exons (Figure S1B), but at the same time had high coverage for *TERT* promoter region, which was completely absent from the OD panel. Furthermore, *EZH2* gene also had remarkably low coverage in OD raw data (Figure S1B) that was probably not sufficient for robust detection of mutations and polymorphisms.

*
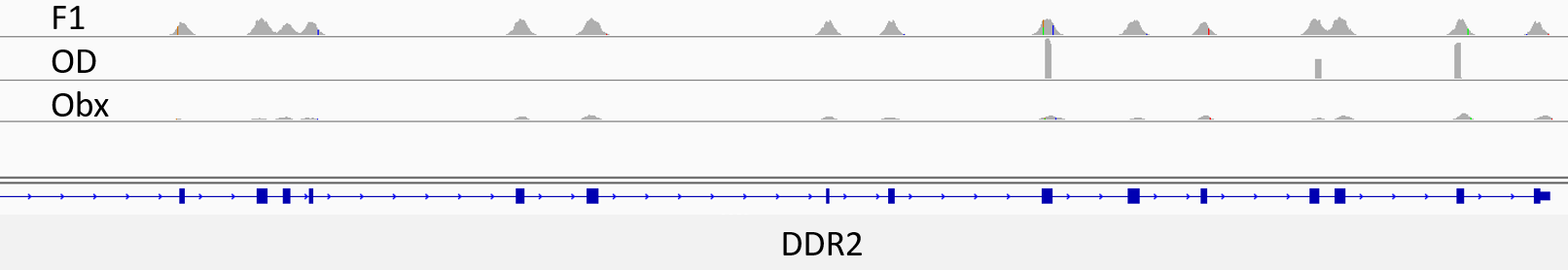
*

*Figure S5. Raw data alignment for gene DDR2using WES-Obx, OD and F1 platforms shown in the same scale settings, peak heights proportionate to sequencing depth(maximum depth set as 3500). Note markedly lower exon coverage and higher apparent sequencing depths for OD platform.*

1. **Additional reads**

Apparently, not all reads obtained by targeted sequencing corresponded to the genes listed in the corresponding F1 of OD reports. Instead, 53% of reads for F1 were ‘off-targets’, which can be estimated as improbably too high proportion of improper reads for the targeted sequencing ( <https://www.illumina.com/documents/products/technotes/technote_optimizing_coverage_for_targeted_resequencing.pdf> ), and may suggest instead the presence of additional not specified targets in the panel. In F1 raw sequencing data, we found reads corresponding to as much as 1739 genes with total coverage above 2000. However, including additional filtering step requiring at least 50% of gene exons having each >100 mapped reads reduced the list of F1 “off-target” genes to 137 items (Figure S6; off-target genes listed in Supplementary file 5).

For the OD platform, only 13% of reads could not be mapped on genes sequences included in the specified gene panel, thus laying in the standard interval for gene enrichment methods.

A
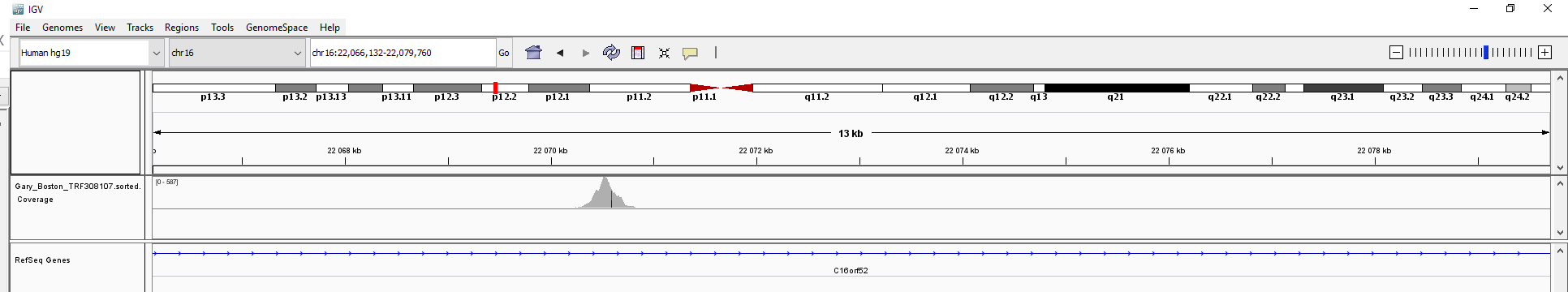


B
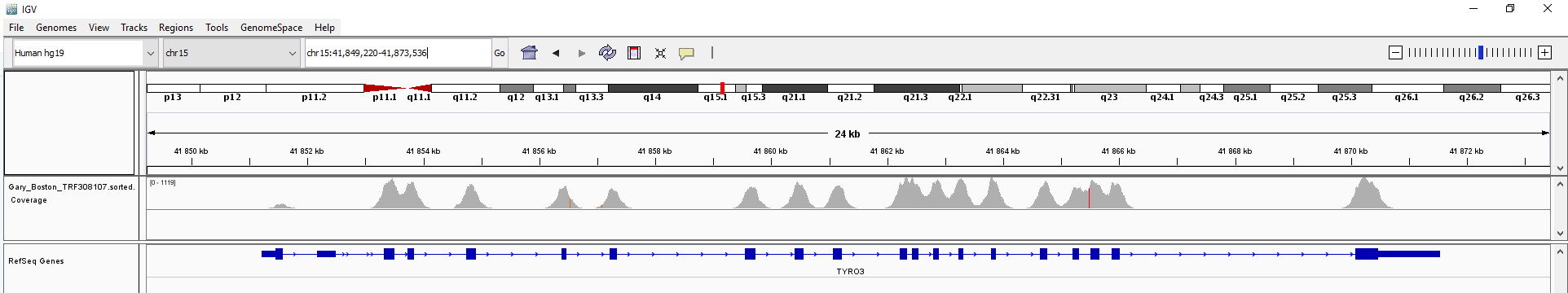


*Figure S6. Mapping of off-target reads obtained using F1 platform. A, an artifact coverage of gene c16orf52 harboring a single peak in intron area. B, coverage of gene TYRO3, which is not listed in F1 panel.*

**4. Comparison of mutations/polymorphisms data obtained using WES-Obx, F1 and OD platforms**

According to reports obtained, in the sample under investigation WES-Obx analysis detected 502 mutations, F1 – sixteen and OD – only two (*PIK3CA, Q546K* and *PTPN11, D61Y*). F1-detected mutations are shown on Table S3, of them those not detected by WES-Obx are highlighted.

*Table S3. Genes presented in F1, but not in Obx report*

| **No** | **Gene/mutation ID** | **Presence in WES-Obx report** | **Presence in F1 report** |
| --- | --- | --- | --- |
| *1* | *MLL2, G1234A* | + | + |
| *2* | *DNMT3A, W297S* | + | + |
| *3* | *PIK3R2, S276N* | + | + |
| *4* | *FANCA, R1053C* | + | + |
| *5* | *RET, E843K* | + | + |
| *6* | *SPTA1, R60** | + | + |
| *7* | *PTPN11, D61Y* | + | + |
| *8* | *PIK3CA, Q546K* | + | + |
| *9* | *FBXW7, D126FS*4* | + | + |
| *10* | *FOXP1, N570S* | + | + |
| *11* | *TSC1, K587R* | + | + |
| *12* | *ABL1, E3A* | - | + |
| *13* | *EPHA5, V647_R648>IL* | - | + |
| *14* | *ZNF217, D323N* | - | + |
| *15* | *AKT3, putative splice site 562-1G>A* | - | + |
| *16* | *IRF4, D33N* | - | + |

Coverage depths (unfiltered reads) for putative mutations detected by the F1 platform but missed in the WES-Obx report are shown on Table S4. As seen from the detailed manual analysis, in three cases (*Znf217*, *Irf4*и*Akt3*) raw WES-Obx data didn’t provide evidence for presence of these mutations. Two other mutations (*Abl1*, *Epha5*) were in fact detectable during manual analysis of the Oncobox raw data but were by some reasons removed from the WES-Obx report, e.g. due to bioinformatic filtering step.

*Table S4. Raw reads coverage of mutations detected by F1, but not WES-Obx platform.*

| **Gene ID** | **WES-Obx** | **F1** |
| --- | --- | --- |
| *Znf217* | C:248, A:1 | C:1400, A:1, T:31 |
| *Irf4* | G:126 | G:716, A:56 |
| ***Abl1*** | **A:315, C:205, G:1** | **A:271, C:252, T:2** |
| *Akt3* | C:510, T:1, A:2 | C:1116, T:148, A:49 |
| ***Epha5*** | **C:390, A:29** | **C:1240, A:158, G1** |

In turn, six mutations in the overlapping gene exons were detected by the WES-Obx but missed in the F1 report (Table S5). For presence of three of these mutations (in genes *Bard1, Ctnna1, Sufu*), F1 dataset had supporting raw data. For three other genes (*Cbl, Ddr2, Fgfr2*) F1 dataset didn’t support presence of mutations. The apparent discrepancy between the platforms may be explained by technical library amplification bias or by different cellular composition of portions of biosamples sent out for sequencing.

*Table S5. Raw reads coverage of mutations detected by Obx, but not F1 platform.*

| **Gene ID** | **WES-Obx** | **F1** |
| --- | --- | --- |
| ***Bard1*** | **C:176, G:170** | **C:716, G:744** |
| ***Ctnna1*** | **C:128, T:126** | **C:458, T:475** |
| ***Sufu*** | **G:139, T:134** | **G:357, T:336** |
| *Cbl* | Mean coverage depth 70, 10 reads with deletion | Mean coverage depth 700, deletion not found |
| *Ddr2* | G:327, A:20 | G:1308, A:2, T:1 |
| *Fgfr2* | Mean coverage depth 380, 17 with deletion | Mean coverage depth 1360, deletion not found |

In the OD panel, there were four genes where both F1 and WES-Obx pipelines detected mutations – *RET, FBXW7, PIK3CA* and *PTPN11*. From these, OD reported the same mutations in only two genes: *PTPN11* and *PIK3CA* but missed the other two (in genes *RET*, *FBXW7*). Our detailed manual raw data analysis revealed that this shortage was because OD platform didn’t sequence gene exons harboring these mutations (Figure S7)


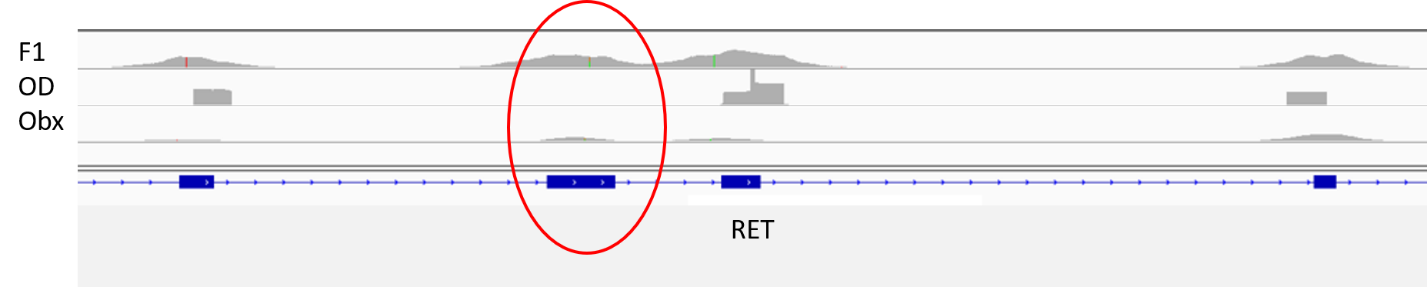


*Figure S7. A fragment of gene RET with coverage profiles of F1, OD and WES-Obx platforms. Red oval marks the exon where mutation was identified by the F1 and WES-Obx platforms but missed in the OD platform. Note the local lack of coverage for OD track.*

1. **Identification of cancer driver mutations**

We then examined alternative allele frequencies to identify most probable cancer driver mutations. This analysis was based on the rationale that they occur at the very beginning of the disease and must be more frequent compared to mutations acquired later during tumor progression. We analyzed frequencies of somatic mutations detected in all three tumor samples (from biopsy, plus surgical samples from stomach and esophageal localizations). The mutations leading to nonsynonymous substitutions, stop-gains or frameshifts were analyzed by PolyPhen2 software to estimate functional changes in protein structures.[1] We found only three potentially driver mutations with frequencies above 20% in most of cancer samples (Figure S8). This left us three respective candidate genes: *PIH1D1* and *FBXW7* with frameshift deletions and *TP53BP1* with stop-gain mutation. Raw reads coverage statistics for these three mutations is shown on Figure S8. Literature search revealed that *FBXW7* is a haploinsufficient tumor suppressor, involved in intestine stem cells fate decision and associated with gastric cancer.[2–4] *TP53BP1* is also a haploinsufficient tumor suppressor in glioma,[5] but also associated with increased risk of gastric cardia adenocarcinoma.[6,7] *PIH1D1* is not known as a tumor suppressor itself but being a part of protein degradation machinery involved in stabilization of p53 and other factors,[8] this gene may have a role in tumorigenesis. The effects of *PIH1D1* haploinsufficiency in cancer are so far unknown.


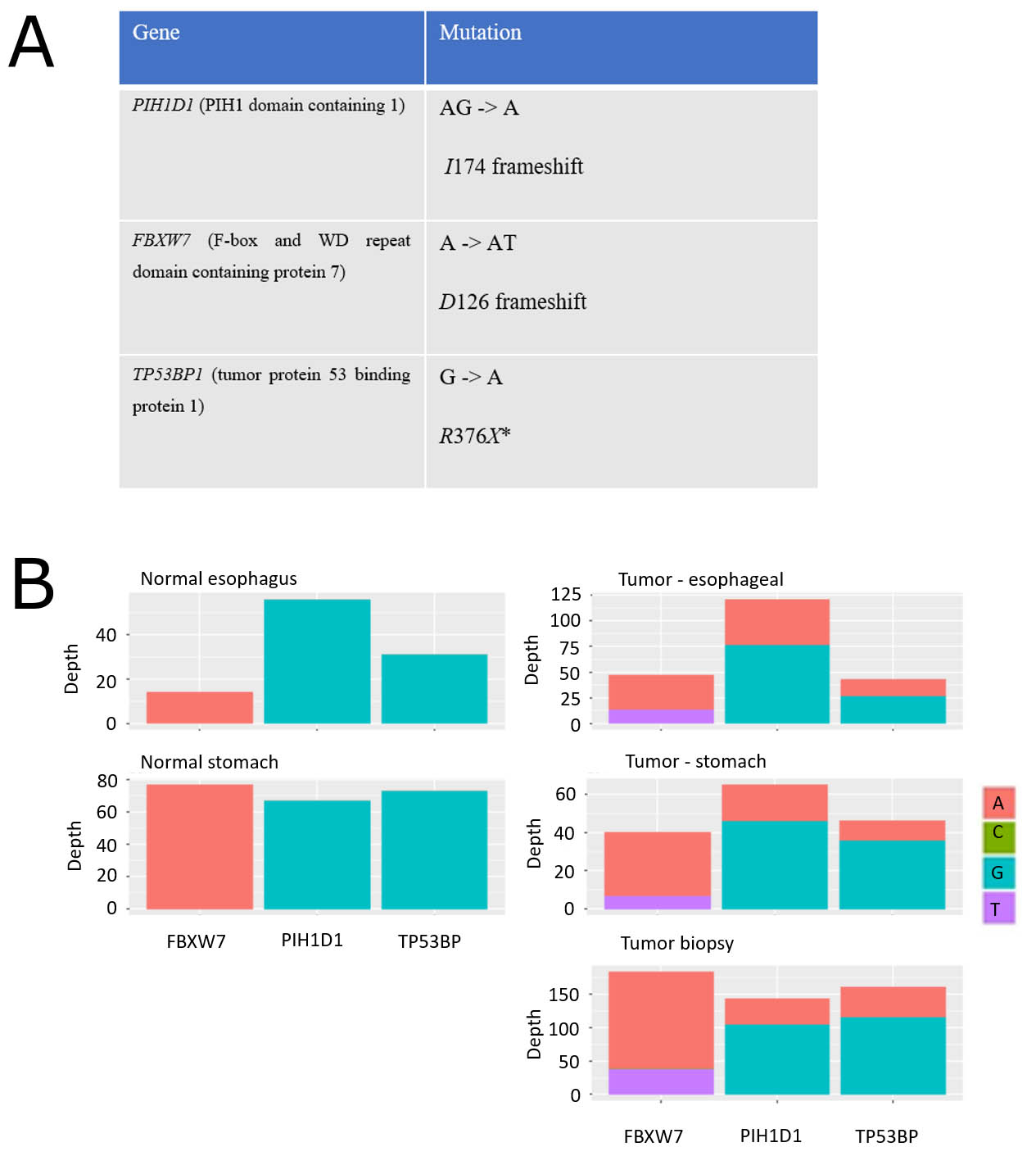


*Figure S8. Potential driver mutation identified. (A) Description of genes with potential driver mutations. (B) Sequencing depth for potential driver mutation sites.*

1. **Copy number variation analysis**

Chromosome aberrations including gene deletions or duplications are common causes of cancer development.[9] The patient biopsy sample for investigated using OncoPanel commercial test, and additional analysis of WES data was done in our laboratory to assess the tumor copy number variations (CNV) profile. To detect CNV we used surgery removed gastric tumor along with the matched normal stomach tissue sample as the control. Stomach samples were used because stomach was a primarily diagnosed tumor localization, therefore matched normal tissue could be directly compared to the tumor. We performed CNV analysis using GATK software.[10] It integrates two types of sample information: coverage ratio between tumor and normal samples and fraction of germline heterozygous alleles. We then compared our results with the available OncoPanel report for the same patient (Figure S9 A,B). The results were highly congruent in both platforms and pointed to major copy number alterations in chromosomes 17 and 20 including *ERBB2*, *BIRC5*, *MMP9* and *SNAI1* genes (copy gains), 19 (almost half of the chromosome duplicated) and X (left arm amplified, while right arm deleted). Copy ratio approach for CNV detection *per se* has a disadvantage that it can’t detect copy-neutral aberrations, e.g. copy-neutral loss of heterozygosity (cnLOH) which is common in cancer biology. To address this, we interrogated cnLOH by analyzing germline heterozygous allele frequencies (Figure S9C). According to this complementary analysis, we found that the whole chromosomes 7, 14 and 15 underwent cnLOH and chromosomes 4, 19 and others underwent partial cnLOH in this cancer case (Figure S9C). Previously, cnLOH of chromosome 4 was reported in up to 50% of cases in gastrointestinal stromal tumor.[11] Moreover, all three driver mutations in genes *FBXW7*, *TP53BP1* and *PIH1D1* identified in our previous analysis were included in the cnLOH areas (chromosome 4, 15 and 19, respectively). Taken together, these findings evidence homozygous deletions of the wild-type alleles of these genes in cancer tissue.


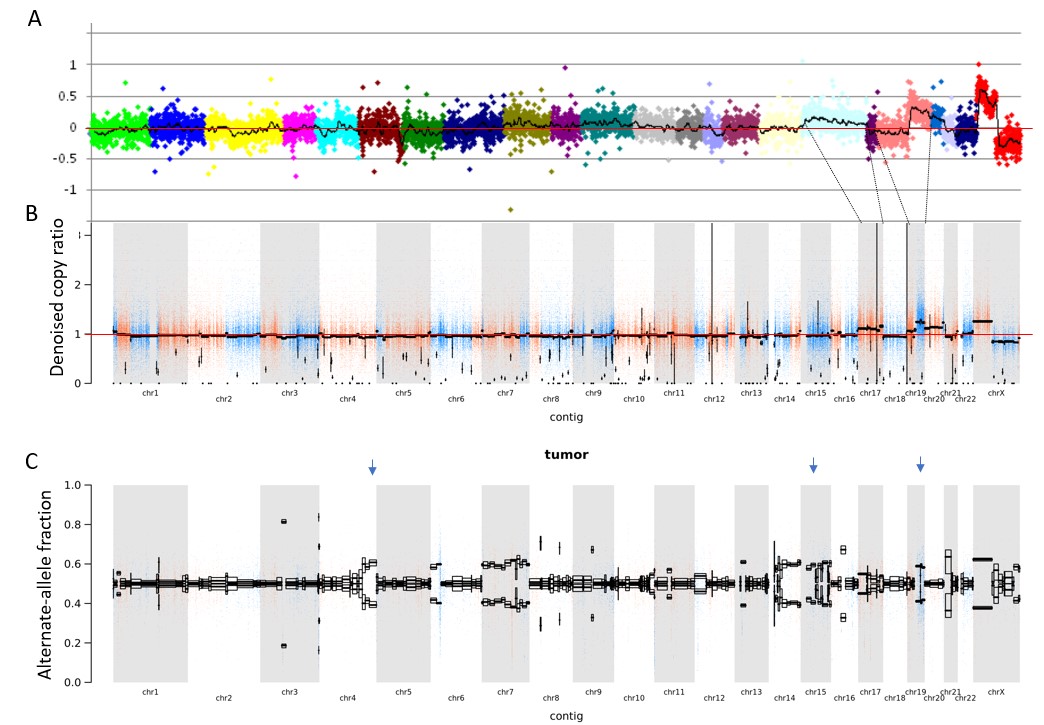


*Figure S9. Copy number variation (CNV) analysis of the gastric cancer case. A: results of CNV analysis included in OncoPanel report. Y axis represents log2 scaled copy ratio between tumor and normal samples. B: our copy ratio analysis, y axis represents log2 scaled copy ratio + 1 between tumor and normal samples. Dashed lines align chromosome positions between panels A and B. C: our alternative allele frequency analysis, arrows represent point mutations in Fbwx7 (chr4), TP53BP1 (chr15) and PIH1D1 (chr19).*

1. **Association of molecular pathway activation and driver mutations**

Three potential driver mutations in genes *FBXW7, TP53BP1* and *PIH1D1* were identified for the patient’s cancer. In clinical context loss of *FBXW7* function may be associated with upregulation of mTOR pathway due to lack of mTOR ubiquitination.[12] At the level of mRNA analysis, we didn’t detect any significant changes in mTOR pathway activity between tumor and normal tissues. On the other hand, FBXW7 also targets for degradation many other oncoproteins such as C-myc, Cyclin E, Notch1 and Jun. Among the top pathways upregulated in cancer we found several that were tightly associated with cell cycle progression, DNA repair and chromosome stability. These pathways could be directly influenced through the candidate driver mutants. For example, the top tumor-upregulated pathway “reactome G0 and Early G1 Pathway” strongly depends on Cyclin E activity that was upregulated in all cancer biosamples. Cyclin E is thought to be one of the major targets of FBXW7.[13] This means that Cyclin E could be upregulated not only at the mRNA, but also at the protein level (Figure S10A). Similarly, we observed upregulation of *BRCA1* and other associated gene products from the “reactome Meiotic recombination Pathway”. BRCA1 acts as antagonist of TP53BP1 protein thus triggering homologous instead of non-homologous end joining repair,[14] (Figure S10B).


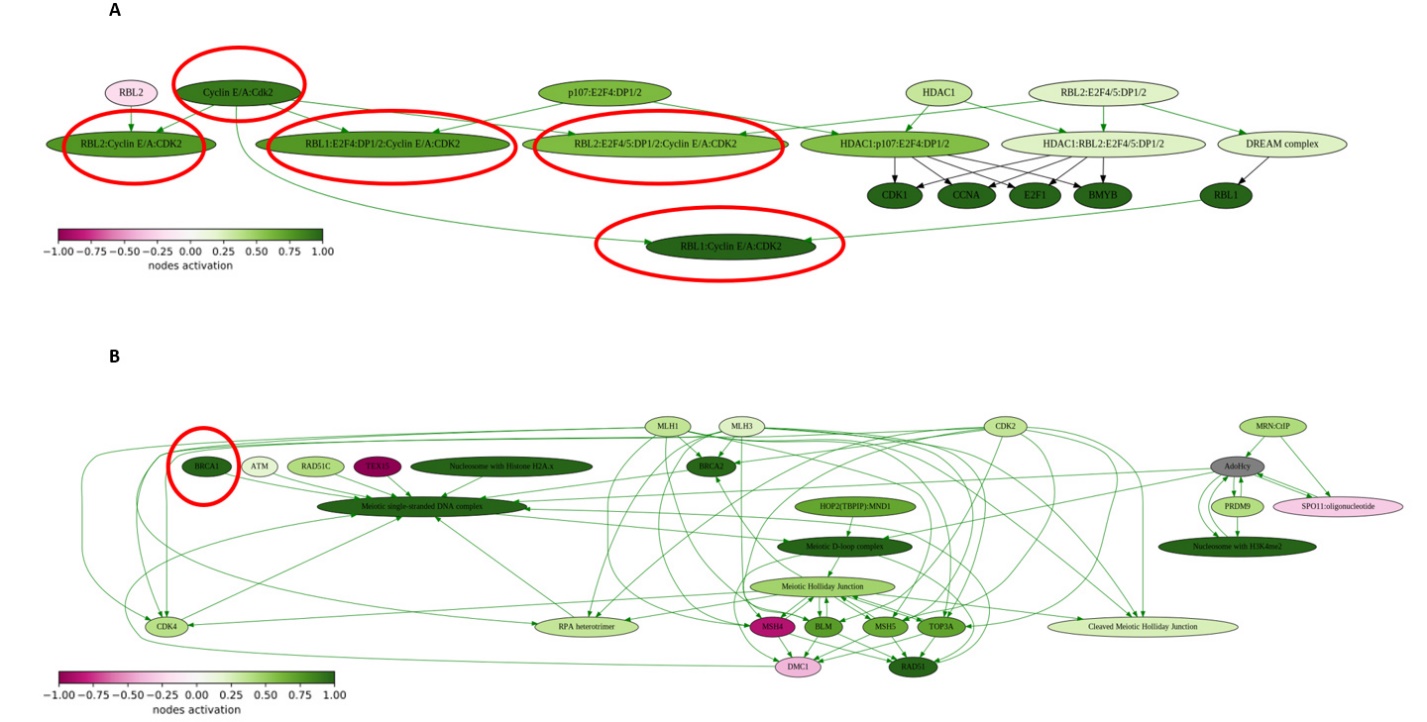


*Figure S10.(A) Pathway activation profile of “Reactome_G0_and_Early_G1_Main_Pathway” in Stomach tumor sample. Cyclin E is targeted for degradation by FBWX7 protein. (B)Pathway activation profile of “Reactome_Meiotic_recombination_Main_Pathway” in Stomach tumor sample. BRCA1 acts as antagonist of TP53BP1 protein, thus triggering homologous end joining repair instead of non-homologous end joining.*

Furthermore, we analyzed occurrences of mutations in genes included in the top differentially regulated pathways. For that purpose, we used 386 mutations identified (Supplementary File 3) as an overlap of all analyzed tumor samples. Topmost significantly up- or downregulated pathways were strongly impacted by patient tumor-specific mutations (Table S6).

*Table S6. Top differentially regulated molecular pathways with highest mutation burden.*

| **Molecular Pathway ID** | **PAL, biopsy** | **PAL, Esophagus tumor** | **PAL, Stomach Tumor** | **PAL, Mean** | **Mutated genes** |
| --- | --- | --- | --- | --- | --- |
| Pathways upregulated in cancer | | | | | |
| Reactome_Immunoregulatory_ interactions_between_ a_Lymphoid_and_a_non-Lymphoid_cell | 0,52 | 0,61 | 0,68 | 0,52 | *KIR2DL4, KIR3DL2* |
| Reactome_Separation_of_Sister_ Chromatids_Main_Pathway | 0,59 | 0,50 | 0,50 | 0,52 | *ANAPC1, PPP2R1B, SPDL1* |
| Reactome_RMTs_methylate_histone_ arginines_Main_Pathway | 0,42 | 0,63 | 0,44 | 0,48 | *ARID1A, DNMT3A, SMARCA2* |
| Pathways downregulated in cancer | | | | | |
| KEGG_Neuroactive_ligand_receptor _interaction_Main_Pathway | -0,27 | -0,34 | -0,19 | -0,27 | *CSH1, GCGR, GRIA2, GRIK5,  HRH4, PRSS2, PRSS3* |
| cAMP_Pathway | -0,20 | -0,23 | -0,15 | -0,20 | *ANAPC1, CAMK2G,  CLCN2, GCGR, RAPGEF3* |
| KEGG_Adrenergic_signaling_in_ cardiomyocytes_Main_Pathway | -0,31 | -0,22 | -0,16 | -0,23 | *ATP2B3, CAMK2G,  PPP2R1B, RAPGEF3* |

MATERIALS AND METHODS

**Tissue sampling**

Primary tumor sampling was done by endoscopy in October 2017. For all molecular studies, the biosamples were stored in the form of formalin fixed, paraffin-embedded (FFPE) tissue blocks. FFPE blocks for primary tumor contained more than 70% of tumor cells according to histological analysis.

Further testing was performed after the residual tumor was resected in March 2018. Samples from gastric and esophageal portions of the lesion were obtained, as well as matching normal tissue samples. According to histological analysis, FFPE blocks for post-operational tumor material contained more than 50% of tumor cells and contained high proportion of infiltrating immune cells. FFPE blocks for matching healthy tissue samples contained histologically normal cells.

**Commercial genetic molecular tests**

Commercial genomic tests were performed by Foundation One (https://www.foundationmedicine.com/genomic-testing), OncoDNA (https://www.oncodna.com/en/) and Dana Farber Institute (Oncopanel; https://www.dana-farber.org/research/featured-research/profile/) using slices of FFPE tissue blocks for primary tumor biopsy.

**Whole exome sequencing and analysis**

DNA was extracted from the FFPE tissue blocks using the AnaPrep FFPE DNA extraction kit following the manufacturer’s instruction. Whole-exome DNA was captured from total genomic DNA using the SeqCap EZ System from NimbleGen according to the manufacturer’s instructions. Briefly, genomic DNA was sheared, size selected to roughly 200-250 base pairs, and the ends were repaired and ligated to specific adapters and multiplexing indexes. Fragments were then incubated with SeqCap biotinylated DNA baits followed by the PCR, and the RNA-DNA hybrids were purified using streptavidin-coated magnetic beads. The RNA baits were then digested to release the targeted DNA fragments, followed by a brief amplification of 15 or less PCR cycles. Sequencing was performed on Illumina Hiseq 3000 for a pair read 150 run. Data quality check was done on Illumina SAV. De-multiplexing was performed with Illumina Bcl2fastq2 v 2.17 program. Mutation calling was performed using Picard and Genome Analysis Toolkit.

**RNA sequencing and gene expression analysis**

RNA extraction was performed using the RecoverAll™ Total Nucleic Acid Isolation Kit for FFPE (Invitrogen) following the manufacturer’s protocol, then RNA Integrity Number (RIN) was measured using Agilent 2100 bio-Analyzer. Agilent RNA 6000 Nano Kit was used to measure RNA concentration. KAPA RNA Hyper with RiboErase kit(Roche) was used for depletion of ribosomal RNA. For library preparations, we used 1-96 KAPA HyperPrep Kit according to the manufacturer’s recommendations. Library concentrations and quality were measured using Qubit ds DNA HS Assay kit (Life Technologies) and Agilent Tapestation (Agilent). RNA sequencing was performed using Illumina HiSeq 3000 equipment for single-end sequencing, 50 bp read length, for approx. 30 million raw reads per each sample. Data quality check was done on Illumina SAV. De-multiplexing was performed with Illumina Bcl2fastq2 v 2.17 program. RNA sequencing FASTQ files were then processed with STAR aligner[15] in “GeneCounts” mode with the Ensembl human transcriptome annotation (Build version GRCh38 and transcript annotation GRCh38.89). Ensembl gene IDs were converted to HGNC gene symbols using Complete HGNC dataset (https://www.genenames.org/, database version from 2017 July 13). In total, expression levels were established for annotated genes having HGNC identifiers.

For comparison of sequencing data obtained using different platforms, initial statistical analysis was implemented using qualimap software (http://qualimap.bioinfo.cipf.es/doc_html/command_line.html). Bed files of genes and exons required for the analysis were obtained using bioMart software (http://www.ensembl.org/biomart/martview/e444c67550be8c04f6b1cc54ae8ca117) and common gene names obtained from the FoundationOne and OncoDNA reports.

**Molecular pathway activation assay and ranking of targeted anticancer drugs**

Patient’s tumor-specific alterations in activation of intracellular molecular pathways in comparison with normal tissues were quantitatively assessed using Oncobox bioinformatical platform[16]. The structures of 3121 molecular pathways were taken from the following public databases: Reactome,[17] NCI Pathway Interaction Database,[18] Kyoto Encyclopedia of Genes and Genomes,[19] HumanCyc,[20] Biocarta,[21] Qiagen (www.qiagen.com/us/shop/genes-and-pathways/pathway-central/). For further pathway analyses, we pre-selected 1748 molecular pathways each including at least 10 gene products.

**References**

1. Adzhubei, I.; Jordan, D.M.; Sunyaev, S.R. Predicting functional effect of human missense mutations using PolyPhen-2. *Curr. Protoc. Hum. Genet.* **2013**, *Chapter 7*, Unit7.20.

2. Jiang, Y.; Qi, X.; Liu, X.; Zhang, J.; Ji, J.; Zhu, Z.; Ren, J.; Yu, Y. Fbxw7 haploinsufficiency loses its protection against DNA damage and accelerates MNU-induced gastric carcinogenesis. *Oncotarget* **2017**, *8*, 33444–33456.

3. Mao, J.-H.; Perez-Losada, J.; Wu, D.; Delrosario, R.; Tsunematsu, R.; Nakayama, K.I.; Brown, K.; Bryson, S.; Balmain, A. Fbxw7/Cdc4 is a p53-dependent, haploinsufficient tumour suppressor gene. *Nature* **2004**, *432*, 775–9.

4. Sancho, R.; Jandke, A.; Davis, H.; Diefenbacher, M.E.; Tomlinson, I.; Behrens, A. F-box and WD repeat domain-containing 7 regulates intestinal cell lineage commitment and is a haploinsufficient tumor suppressor. *Gastroenterology* **2010**, *139*, 929–41.

5. Squatrito, M.; Vanoli, F.; Schultz, N.; Jasin, M.; Holland, E.C. 53BP1 is a haploinsufficient tumor suppressor and protects cells from radiation response in glioma. *Cancer Res.* **2012**, *72*, 5250–60.

6. Zhang, S.; Tang, W.; Ding, G.; Liu, C.; Liu, R.; Chen, S.; Gu, H.; Yu, C. Variant TP53BP1 rs560191 G&gt;C is associated with risk of gastric cardia adenocarcinoma in a Chinese Han population. *Chin. J. Cancer Res.* **2015**, *27*, 156–62.

7. Shao, A.; Zheng, L.; Chen, S.; Gu, H.; Jing, H. *p21, p53, TP53BP1* and *p73* polymorphisms and the risk of gastric cardia adenocarcinoma in a Chinese population. *Biomarkers* **2015**, *20*, 109–115.

8. Hořejší, Z.; Stach, L.; Flower, T.G.; Joshi, D.; Flynn, H.; Skehel, J.M.; O’Reilly, N.J.; Ogrodowicz, R.W.; Smerdon, S.J.; Boulton, S.J. Phosphorylation-dependent PIH1D1 interactions define substrate specificity of the R2TP cochaperone complex. *Cell Rep.* **2014**, *7*, 19–26.

9. Hudler, P. Genetic aspects of gastric cancer instability. *ScientificWorldJournal.* **2012**, *2012*, 761909.

10. Van der Auwera, G.A.; Carneiro, M.O.; Hartl, C.; Poplin, R.; Del Angel, G.; Levy-Moonshine, A.; Jordan, T.; Shakir, K.; Roazen, D.; Thibault, J.; et al. From FastQ data to high confidence variant calls: the Genome Analysis Toolkit best practices pipeline. *Curr. Protoc. Bioinforma.* **2013**, *43*, 11.10.1-33.

11. Lourenço, N.; Hélias-Rodzewicz, Z.; Bachet, J.-B.; Brahimi-Adouane, S.; Jardin, F.; Tran van Nhieu, J.; Peschaud, F.; Martin, E.; Beauchet, A.; Chibon, F.; et al. Copy-neutral loss of heterozygosity and chromosome gains and losses are frequent in gastrointestinal stromal tumors. *Mol. Cancer* **2014**, *13*, 246.

12. Yeh, C.-H.; Bellon, M.; Nicot, C. FBXW7: a critical tumor suppressor of human cancers. *Mol. Cancer* **2018**, *17*, 115.

13. Sailo, B.L.; Banik, K.; Girisa, S.; Bordoloi, D.; Fan, L.; Halim, C.E.; Wang, H.; Kumar, A.P.; Zheng, D.; Mao, X.; et al. FBXW7 in Cancer: What Has Been Unraveled Thus Far? *Cancers (Basel).* **2019**, *11*, 246.

14. Isono, M.; Niimi, A.; Oike, T.; Hagiwara, Y.; Sato, H.; Sekine, R.; Yoshida, Y.; Isobe, S.-Y.; Obuse, C.; Nishi, R.; et al. BRCA1 Directs the Repair Pathway to Homologous Recombination by Promoting 53BP1 Dephosphorylation. *Cell Rep.* **2017**, *18*, 520–532.

15. Dobin, A.; Davis, C.A.; Schlesinger, F.; Drenkow, J.; Zaleski, C.; Jha, S.; Batut, P.; Chaisson, M.; Gingeras, T.R. STAR: ultrafast universal RNA-seq aligner. *Bioinformatics* **2013**, *29*, 15–21.

16. Garazha A, B.A.B.N.S.M. Quantitation of molecular pathway activation using RNA sequencing data. *Methods Mol. Biol.* **2019**, In press.

17. Croft, D.; Mundo, A.F.; Haw, R.; Milacic, M.; Weiser, J.; Wu, G.; Caudy, M.; Garapati, P.; Gillespie, M.; Kamdar, M.R.; et al. The Reactome pathway knowledgebase. *Nucleic Acids Res.* **2014**, *42*, D472–D477.

18. Schaefer, C.F.; Anthony, K.; Krupa, S.; Buchoff, J.; Day, M.; Hannay, T.; Buetow, K.H. PID: the Pathway Interaction Database. *Nucleic Acids Res.* **2009**, *37*, D674-9.

19. Nakaya, A.; Katayama, T.; Itoh, M.; Hiranuka, K.; Kawashima, S.; Moriya, Y.; Okuda, S.; Tanaka, M.; Tokimatsu, T.; Yamanishi, Y.; et al. KEGG OC: a large-scale automatic construction of taxonomy-based ortholog clusters. *Nucleic Acids Res.* **2013**, *41*, D353-7.

20. Romero, P.; Wagg, J.; Green, M.L.; Kaiser, D.; Krummenacker, M.; Karp, P.D. Computational prediction of human metabolic pathways from the complete human genome. *Genome Biol.* **2004**, *6*, R2.

21. Nishimura, D. BioCarta. *Biotech Softw. Internet Rep.* **2001**, *2*, 117–120.

22. Borisov, N.; Sorokin, M.; Garazha, A.; Buzdin, A. Quantitation of Molecular Pathway Activation Using RNA Sequencing Data. *Methods Mol. Biol.* **2020**, *2063*, 189–206.
