# Supplementary material for: Transcriptomic and Genomic Testing to Guide Individualized Treatment in Chemoresistant Gastric Cancer Case
Source: Biomedicines. 2020 Mar 23;8(3):67. doi: 10.3390/biomedicines8030067 (PMC7148467; doi:10.3390/biomedicines8030067)
Supplement: Supplementary file 1 [file biomedicines-08-00067-s001.zip › biomedicines-742411-supplementary/Supplementary file 2.docx]

List of mutation reported by Obx based on biopsy WES, no normalization was used

| # | Gene | Nucleotide change | Amino Acid change | Type |
| --- | --- | --- | --- | --- |
| 1 | CSAG1 | c.G185A | p.R62K | nonsynonymous SNV |
| 2 | USP6 | c.G319T | p.G107C | nonsynonymous SNV |
| 3 | PDCD2L | c.G965A | p.G322E | nonsynonymous SNV |
| 4 | ALG1 | c.C1182G | p.F394L | nonsynonymous SNV |
| 5 | GFY | c.C719A | p.T240N | nonsynonymous SNV |
| 6 | FBXO36 | c.A68G | p.Y23C | nonsynonymous SNV |
| 7 | KIR3DL3 | c.T971C | p.V324A | nonsynonymous SNV |
| 8 | OR4A47 | c.T706C | p.S236P | nonsynonymous SNV |
| 9 | TCF15 | c.G568A | p.G190R | nonsynonymous SNV |
| 10 | ZNF880 | c.G1213A | p.E405K | nonsynonymous SNV |
| 11 | DNTTIP2 | c.G1430A | p.G477E | nonsynonymous SNV |
| 12 | AMZ2 | c.C64G | p.L22V | nonsynonymous SNV |
| 13 | ZNF543 | c.G1675A | p.G559R | nonsynonymous SNV |
| 14 | CEACAM6 | c.T716G | p.V239G | nonsynonymous SNV |
| 15 | LCN6 | c.T40C | p.S14P | nonsynonymous SNV |
| 16 | PCDHGA10 | c.G2221A | p.G741S | nonsynonymous SNV |
| 17 | PCDHA1 | c.A1345C | p.N449H | nonsynonymous SNV |
| 18 | C1orf109 | c.G266A | p.G89E | nonsynonymous SNV |
| 19 | KIAA1671 | c.A1316G | p.K439R | nonsynonymous SNV |
| 20 | ZNF285 | c.G1141A | p.G381R | nonsynonymous SNV |
| 21 | SCN4A | c.C968T | p.T323M | nonsynonymous SNV |
| 22 | NEBL | c.G180C | p.K60N | nonsynonymous SNV |
| 23 | NBPF11 | c.A320G | p.N107S | nonsynonymous SNV |
| 24 | USP17L7 | c.T2C | p.M1T | nonsynonymous SNV |
| 25 | LOC729159 | c.C295T | p.P99S | nonsynonymous SNV |
| 26 | LILRB2 | c.T483A | p.D161E | nonsynonymous SNV |
| 27 | RPL29 | c.385_386insCCAAGG | p.A129delinsAKA | nonframeshift insertion |
| 28 | RNF157 | c.C416T | p.T139M | nonsynonymous SNV |
| 29 | IFI16 | c.C1325T | p.T442I | nonsynonymous SNV |
| 30 | MRGPRX4 | c.C806T | p.P269L | nonsynonymous SNV |
| 31 | TTN | c.C98075G | p.T32692R | nonsynonymous SNV |
| 32 | PCDHB8 | c.C2299T | p.L767F | nonsynonymous SNV |
| 33 | TTC13 | c.C602G | p.A201G | nonsynonymous SNV |
| 34 | FOXP1 | c.A1709G | p.N570S | nonsynonymous SNV |
| 35 | CUTA | c.T76C | p.S26P | nonsynonymous SNV |
| 36 | HERC2 | c.C5040G | p.S1680R | nonsynonymous SNV |
| 37 | DNAH7 | c.4787dupA | p.Y1596_E1597delinsX | stopgain |
| 38 | CDK10 | c.G1073A | p.C358Y | nonsynonymous SNV |
| 39 | FTMT | c.G646C | p.V216L | nonsynonymous SNV |
| 40 | NLRP2 | c.A991G | p.I331V | nonsynonymous SNV |
| 41 | UHRF1 | c.G718C | p.D240H | nonsynonymous SNV |
| 42 | EFL1 | c.C2929T | p.P977S | nonsynonymous SNV |
| 43 | GFM1 | c.A127G | p.N43D | nonsynonymous SNV |
| 44 | MED30 | c.G319C | p.D107H | nonsynonymous SNV |
| 45 | CDH19 | c.A977C | p.H326P | nonsynonymous SNV |
| 46 | STPG4 | c.A185G | p.E62G | nonsynonymous SNV |
| 47 | ZNF180 | c.C815G | p.S272C | nonsynonymous SNV |
| 48 | CCDC190 | c.G602T | p.S201I | nonsynonymous SNV |
| 49 | TTLL1 | c.A53G | p.N18S | nonsynonymous SNV |
| 50 | POU6F1 | c.A280C | p.T94P | nonsynonymous SNV |
| 51 | RNF213 | c.C14213T | p.S4738F | nonsynonymous SNV |
| 52 | MAGEF1 | c.470_471insGGA | p.E157delinsEE | nonframeshift insertion |
| 53 | AASDH | c.A1241G | p.D414G | nonsynonymous SNV |
| 54 | PLK1 | c.G1091A | p.R364Q | nonsynonymous SNV |
| 55 | TAS2R3 | c.A536G | p.Y179C | nonsynonymous SNV |
| 56 | RALGPS2 | c.A628T | p.T210S | nonsynonymous SNV |
| 57 | TCHH | c.1499_1500insGGAGAGGCGCGAGCAGCA | p.Q500delinsQERREQQ | nonframeshift insertion |
| 58 | CRYBG1 | c.C3494T | p.T1165M | nonsynonymous SNV |
| 59 | AIPL1 | c.C1108T | p.P370S | nonsynonymous SNV |
| 60 | TAF2 | c.A157G | p.I53V | nonsynonymous SNV |
| 61 | GOLGA4 | c.A4678G | p.T1560A | nonsynonymous SNV |
| 62 | VPS37A | c.C700A | p.L234I | nonsynonymous SNV |
| 63 | TSPAN10 | c.794_795insTAAC | p.F265fs | frameshift insertion |
| 64 | IFI27 | c.122_125del | p.V41fs | frameshift deletion |
| 65 | ZNF43 | c.C2357A | p.P786H | nonsynonymous SNV |
| 66 | RNMT | c.G58C | p.A20P | nonsynonymous SNV |
| 67 | LRP6 | c.G3130A | p.D1044N | nonsynonymous SNV |
| 68 | PTK7 | c.G19A | p.G7R | nonsynonymous SNV |
| 69 | SPTY2D1 | c.A301G | p.I101V | nonsynonymous SNV |
| 70 | NBPF15 | c.G1763A | p.C588Y | nonsynonymous SNV |
| 71 | PCDHB16 | c.G1243C | p.A415P | nonsynonymous SNV |
| 72 | ARL9 | c.G38A | p.R13Q | nonsynonymous SNV |
| 73 | CEACAM7 | c.A358T | p.I120F | nonsynonymous SNV |
| 74 | KAZN | c.G1566T | p.E522D | nonsynonymous SNV |
| 75 | PCDHB9 | c.T716C | p.V239A | nonsynonymous SNV |
| 76 | TBCE | c.C214T | p.P72S | nonsynonymous SNV |
| 77 | TSPAN19 | c.A49G | p.I17V | nonsynonymous SNV |
| 78 | C2orf71 | c.C1461G | p.S487R | nonsynonymous SNV |
| 79 | DNAH14 | c.A1951G | p.I651V | nonsynonymous SNV |
| 80 | ZNF681 | c.C184A | p.P62T | nonsynonymous SNV |
| 81 | NEK10 | c.G1094A | p.R365Q | nonsynonymous SNV |
| 82 | COL4A6 | c.1971_1972insAAG | p.V658delinsKV | nonframeshift insertion |
| 83 | FANCM | c.A624G | p.I208M | nonsynonymous SNV |
| 84 | PHYKPL | c.G88C | p.D30H | nonsynonymous SNV |
| 85 | PCDHA13 | c.G1636A | p.G546S | nonsynonymous SNV |
| 86 | TRPM2 | c.G2386A | p.V796M | nonsynonymous SNV |
| 87 | CCL23 | c.G341A | p.R114Q | nonsynonymous SNV |
| 88 | CEACAM5 | c.G1192A | p.E398K | nonsynonymous SNV |
| 89 | TTC25 | c.C1390T | p.Q464X | stopgain |
| 90 | PLCXD1 | c.T512C | p.I171T | nonsynonymous SNV |
| 91 | ZNF841 | c.C1031T | p.P344L | nonsynonymous SNV |
| 92 | CCDC34 | c.G313A | p.A105T | nonsynonymous SNV |
| 93 | HMGXB3 | c.T1750A | p.S584T | nonsynonymous SNV |
| 94 | NEPRO | c.G397A | p.E133K | nonsynonymous SNV |
| 95 | NOC3L | c.A134G | p.E45G | nonsynonymous SNV |
| 96 | CCDC180 | c.C3419T | p.T1140I | nonsynonymous SNV |
| 97 | LAD1 | c.C116T | p.S39F | nonsynonymous SNV |
| 98 | DCAF8L2 | c.G193T | p.D65Y | nonsynonymous SNV |
| 99 | TP53BP2 | c.C268T | p.R90C | nonsynonymous SNV |
| 100 | FANCA | c.A4252T | p.T1418S | nonsynonymous SNV |
| 101 | FASN | c.G6374A | p.R2125Q | nonsynonymous SNV |
| 102 | NLRP13 | c.A878G | p.D293G | nonsynonymous SNV |
| 103 | SORBS1 | c.G3422A | p.G1141E | nonsynonymous SNV |
| 104 | VSIG4 | c.G274T | p.V92F | nonsynonymous SNV |
| 105 | HEATR4 | c.C488A | p.P163H | nonsynonymous SNV |
| 106 | MIER3 | c.C1193T | p.A398V | nonsynonymous SNV |
| 107 | SMURF2 | c.A314G | p.N105S | nonsynonymous SNV |
| 108 | CCNY | c.G800A | p.R267K | nonsynonymous SNV |
| 109 | NOC3L | c.G1110A | p.M370I | nonsynonymous SNV |
| 110 | MYO7B | c.T6061G | p.S2021A | nonsynonymous SNV |
| 111 | DDX18 | c.G1111A | p.V371I | nonsynonymous SNV |
| 112 | MALRD1 | c.C1997T | p.A666V | nonsynonymous SNV |
| 113 | SAMD3 | c.C1112T | p.T371I | nonsynonymous SNV |
| 114 | ZNF638 | c.C1483T | p.R495C | nonsynonymous SNV |
| 115 | MDP1 | c.G508T | p.G170W | nonsynonymous SNV |
| 116 | PLEKHA3 | c.T851C | p.M284T | nonsynonymous SNV |
| 117 | ITGA1 | c.C3363A | p.S1121R | nonsynonymous SNV |
| 118 | PHC3 | c.A2233G | p.K745E | nonsynonymous SNV |
| 119 | OR5B12 | c.G911A | p.G304E | nonsynonymous SNV |
| 120 | ANKS6 | c.T2564C | p.F855S | nonsynonymous SNV |
| 121 | MCCC1 | c.G535A | p.E179K | nonsynonymous SNV |
| 122 | ZNF343 | c.A743G | p.N248S | nonsynonymous SNV |
| 123 | CHRNA2 | c.C840A | p.D280E | nonsynonymous SNV |
| 124 | FBLIM1 | c.G931A | p.G311R | nonsynonymous SNV |
| 125 | PHLPP2 | c.G2756A | p.S919N | nonsynonymous SNV |
| 126 | PTPRD | c.G2552A | p.G851E | nonsynonymous SNV |
| 127 | TGS1 | c.A478G | p.I160V | nonsynonymous SNV |
| 128 | SLC25A17 | c.G659A | p.R220Q | nonsynonymous SNV |
| 129 | GPRC6A | c.C2771T | p.S924L | nonsynonymous SNV |
| 130 | NAV3 | c.G1529A | p.S510N | nonsynonymous SNV |
| 131 | IL36B | c.A328G | p.I110V | nonsynonymous SNV |
| 132 | ADAMTS16 | c.G2366A | p.R789H | nonsynonymous SNV |
| 133 | ARHGAP19 | c.C1099T | p.H367Y | nonsynonymous SNV |
| 134 | TCERG1L | c.G1721A | p.R574Q | nonsynonymous SNV |
| 135 | ZBED4 | c.T2092G | p.S698A | nonsynonymous SNV |
| 136 | MYH3 | c.C3008T | p.A1003V | nonsynonymous SNV |
| 137 | OR6C6 | c.A123C | p.L41F | nonsynonymous SNV |
| 138 | SLC2A6 | c.G283A | p.G95R | nonsynonymous SNV |
| 139 | MISP | c.G295A | p.A99T | nonsynonymous SNV |
| 140 | PLBD1 | c.G1313A | p.R438Q | nonsynonymous SNV |
| 141 | TTN | c.A36509T | p.E12170V | nonsynonymous SNV |
| 142 | SACS | c.C8393A | p.P2798Q | nonsynonymous SNV |
| 143 | AHNAK | c.G2917A | p.G973R | nonsynonymous SNV |
| 144 | ETFBKMT | c.C583G | p.L195V | nonsynonymous SNV |
| 145 | CHD7 | c.2049_2050insAAAGCA | p.K683delinsKKA | nonframeshift insertion |
| 146 | SBK3 | c.C41G | p.P14R | nonsynonymous SNV |
| 147 | ZNF134 | c.A312C | p.Q104H | nonsynonymous SNV |
| 148 | SRBD1 | c.A2567T | p.Q856L | nonsynonymous SNV |
| 149 | ZNF615 | c.A617G | p.Q206R | nonsynonymous SNV |
| 150 | CXCL10 | c.C85T | p.R29C | nonsynonymous SNV |
| 151 | ALKBH1 | c.G405A | p.M135I | nonsynonymous SNV |
| 152 | BOD1L1 | c.G4759C | p.A1587P | nonsynonymous SNV |
| 153 | DNAH6 | c.A2179G | p.T727A | nonsynonymous SNV |
| 154 | PDIA2 | c.A347G | p.E116G | nonsynonymous SNV |
| 155 | ANK1 | c.A4096G | p.M1366V | nonsynonymous SNV |
| 156 | FRMPD2 | c.G1051A | p.G351R | nonsynonymous SNV |
| 157 | MYH3 | c.G5254A | p.A1752T | nonsynonymous SNV |
| 158 | ANKRD34B | c.C70T | p.R24C | nonsynonymous SNV |
| 159 | PGLYRP4 | c.557dupA | p.Y186_V187delinsX | stopgain |
| 160 | MUC5AC | c.8019_8020insACCAGCACAACTTCTGCTTCTACA | p.T2673delinsTTSTTSAST | nonframeshift insertion |
| 161 | SRP68 | c.A808G | p.T270A | nonsynonymous SNV |
| 162 | GARNL3 | c.G1921A | p.A641T | nonsynonymous SNV |
| 163 | HOXD8 | c.C8T | p.P3L | nonsynonymous SNV |
| 164 | GXYLT2 | c.A356G | p.N119S | nonsynonymous SNV |
| 165 | ASH1L | c.T1937A | p.I646K | nonsynonymous SNV |
| 166 | SUSD2 | c.T328A | p.S110T | nonsynonymous SNV |
| 167 | PLEC | c.G8592C | p.E2864D | nonsynonymous SNV |
| 168 | NLRP7 | c.A1532G | p.K511R | nonsynonymous SNV |
| 169 | SLC7A6 | c.A1250G | p.K417R | nonsynonymous SNV |
| 170 | PKHD1L1 | c.G3434C | p.G1145A | nonsynonymous SNV |
| 171 | TMEM243 | c.C189A | p.F63L | nonsynonymous SNV |
| 172 | GON4L | c.C2119G | p.L707V | nonsynonymous SNV |
| 173 | DMXL1 | c.A4765G | p.M1589V | nonsynonymous SNV |
| 174 | INO80E | c.C299T | p.P100L | nonsynonymous SNV |
| 175 | GSE1 | c.1024_1035del | p.342_345del | nonframeshift deletion |
| 176 | HCFC1 | c.G2626A | p.G876S | nonsynonymous SNV |
| 177 | TDRD15 | c.C1237G | p.L413V | nonsynonymous SNV |
| 178 | DBR1 | c.G1282C | p.E428Q | nonsynonymous SNV |
| 179 | PGS1 | c.G1513A | p.V505M | nonsynonymous SNV |
| 180 | SPTBN5 | c.G4100C | p.R1367T | nonsynonymous SNV |
| 181 | LYG2 | c.A445G | p.T149A | nonsynonymous SNV |
| 182 | PSME1 | c.A149G | p.N50S | nonsynonymous SNV |
| 183 | ACTB | c.G530A | p.R177H | nonsynonymous SNV |
| 184 | GOLGA6L10 | c.A638G | p.E213G | nonsynonymous SNV |
| 185 | IGIP | c.A52G | p.T18A | nonsynonymous SNV |
| 186 | SLC9A7 | c.A1327G | p.I443V | nonsynonymous SNV |
| 187 | CCDC191 | c.C653T | p.T218I | nonsynonymous SNV |
| 188 | NCAPH2 | c.G1147C | p.G383R | nonsynonymous SNV |
| 189 | P4HTM | c.A679G | p.I227V | nonsynonymous SNV |
| 190 | NT5DC2 | c.C193T | p.R65C | nonsynonymous SNV |
| 191 | JRK | c.A1426G | p.R476G | nonsynonymous SNV |
| 192 | RET | c.G2527A | p.E843K | nonsynonymous SNV |
| 193 | RIBC2 | c.A370G | p.K124E | nonsynonymous SNV |
| 194 | PKD1L1 | c.C4408T | p.R1470W | nonsynonymous SNV |
| 195 | CSF3R | c.G2503A | p.E835K | nonsynonymous SNV |
| 196 | CCDC77 | c.C913T | p.L305F | nonsynonymous SNV |
| 197 | SLC35D3 | c.G951C | p.Q317H | nonsynonymous SNV |
| 198 | SLC2A6 | c.G391C | p.G131R | nonsynonymous SNV |
| 199 | AIFM2 | c.A469G | p.M157V | nonsynonymous SNV |
| 200 | MUC4 | c.G5305C | p.V1769L | nonsynonymous SNV |
| 201 | WDPCP | c.G1333C | p.A445P | nonsynonymous SNV |
| 202 | B4GALNT2 | c.C1567T | p.R523W | nonsynonymous SNV |
| 203 | TNFSF13 | c.G13C | p.V5L | nonsynonymous SNV |
| 204 | AVPR1B | c.G195C | p.K65N | nonsynonymous SNV |
| 205 | DNAH17 | c.G10066A | p.V3356I | nonsynonymous SNV |
| 206 | PARD3 | c.G875C | p.S292T | nonsynonymous SNV |
| 207 | TBC1D4 | c.A3617G | p.N1206S | nonsynonymous SNV |
| 208 | ENPP7 | c.C11T | p.P4L | nonsynonymous SNV |
| 209 | GAS2L2 | c.C2252A | p.A751D | nonsynonymous SNV |
| 210 | ZNF180 | c.C267G | p.C89W | nonsynonymous SNV |
| 211 | ADGRD1 | c.C890A | p.S297Y | nonsynonymous SNV |
| 212 | PPFIA1 | c.C1958A | p.T653K | nonsynonymous SNV |
| 213 | VSIG4 | c.C1148T | p.T383I | nonsynonymous SNV |
| 214 | PCDHA7 | c.G1027A | p.V343I | nonsynonymous SNV |
| 215 | SYNE1 | c.A22516G | p.S7506G | nonsynonymous SNV |
| 216 | SERINC3 | c.C1286T | p.P429L | nonsynonymous SNV |
| 217 | ANKZF1 | c.G439A | p.E147K | nonsynonymous SNV |
| 218 | GABRQ | c.A1432T | p.I478F | nonsynonymous SNV |
| 219 | LETM1 | c.A1760G | p.K587R | nonsynonymous SNV |
| 220 | AMPD1 | c.A1694T | p.E565V | nonsynonymous SNV |
| 221 | TMEM132D | c.A1661G | p.D554G | nonsynonymous SNV |
| 222 | KIF2A | c.T1732A | p.Y578N | nonsynonymous SNV |
| 223 | IL6ST | c.G1495A | p.V499I | nonsynonymous SNV |
| 224 | FAXDC2 | c.T437C | p.M146T | nonsynonymous SNV |
| 225 | PAPLN | c.C2456T | p.A819V | nonsynonymous SNV |
| 226 | ADGB | c.A1664G | p.H555R | nonsynonymous SNV |
| 227 | GINS2 | c.A71G | p.D24G | nonsynonymous SNV |
| 228 | DPH1 | c.C746G | p.P249R | nonsynonymous SNV |
| 229 | PRRC2B | c.G238A | p.V80I | nonsynonymous SNV |
| 230 | FNDC7 | c.G161C | p.G54A | nonsynonymous SNV |
| 231 | BRAP | c.C1672A | p.Q558K | nonsynonymous SNV |
| 232 | SMIM24 | c.C200T | p.T67I | nonsynonymous SNV |
| 233 | FANCA | c.C3157T | p.R1053C | nonsynonymous SNV |
| 234 | TTC24 | c.G1052C | p.G351A | nonsynonymous SNV |
| 235 | CD93 | c.G1097A | p.R366H | nonsynonymous SNV |
| 236 | MUC4 | c.8810_8818del | p.2937_2940del | nonframeshift deletion |
| 237 | ENDOU | c.G103T | p.D35Y | nonsynonymous SNV |
| 238 | PRPF39 | c.A649G | p.N217D | nonsynonymous SNV |
| 239 | HOXB13 | c.C720A | p.F240L | nonsynonymous SNV |
| 240 | NUBP2 | c.G448A | p.G150S | nonsynonymous SNV |
| 241 | LMF2 | c.C976T | p.L326F | nonsynonymous SNV |
| 242 | C8orf74 | c.T698G | p.L233R | nonsynonymous SNV |
| 243 | TAS2R1 | c.G505A | p.E169K | nonsynonymous SNV |
| 244 | NLRP2 | c.1424_1425insCTA | p.C475delinsCY | nonframeshift insertion |
| 245 | PIK3R2 | c.G827A | p.S276N | nonsynonymous SNV |
| 246 | ELP2 | c.C2591T | p.A864V | nonsynonymous SNV |
| 247 | MIA2 | c.C646T | p.P216S | nonsynonymous SNV |
| 248 | C1orf116 | c.A1757G | p.N586S | nonsynonymous SNV |
| 249 | GATAD2A | c.G880A | p.V294I | nonsynonymous SNV |
| 250 | SLC4A9 | c.G1883A | p.R628H | nonsynonymous SNV |
| 251 | FLII | c.G3728A | p.R1243H | nonsynonymous SNV |
| 252 | STIM1 | c.G1828A | p.A610T | nonsynonymous SNV |
| 253 | NSUN4 | c.G968A | p.G323D | nonsynonymous SNV |
| 254 | FCAMR | c.C487T | p.H163Y | nonsynonymous SNV |
| 255 | NCOA4 | c.T22G | p.F8V | nonsynonymous SNV |
| 256 | GRIA2 | c.G2366T | p.C789F | nonsynonymous SNV |
| 257 | THAP11 | c.C523G | p.P175A | nonsynonymous SNV |
| 258 | MC5R | c.C914T | p.T305I | nonsynonymous SNV |
| 259 | CDH23 | c.G8053T | p.A2685S | nonsynonymous SNV |
| 260 | DNAH14 | c.A10597G | p.M3533V | nonsynonymous SNV |
| 261 | SPATC1 | c.C875T | p.T292I | nonsynonymous SNV |
| 262 | LMAN1L | c.G1441A | p.V481M | nonsynonymous SNV |
| 263 | NEK11 | c.G1525C | p.E509Q | nonsynonymous SNV |
| 264 | LUZP4 | c.824_825insCACTCAGAGAGATCTCGTGGC | p.V275delinsVTQRDLVA | nonframeshift insertion |
| 265 | EXOC3L4 | c.G1937A | p.R646Q | nonsynonymous SNV |
| 266 | COQ8A | c.A1409G | p.N470S | nonsynonymous SNV |
| 267 | ANKZF1 | c.C1370T | p.T457I | nonsynonymous SNV |
| 268 | XIRP2 | c.G3484A | p.V1162I | nonsynonymous SNV |
| 269 | KIAA1210 | c.C508A | p.L170I | nonsynonymous SNV |
| 270 | PYGL | c.C1145T | p.P382L | nonsynonymous SNV |
| 271 | CACNA1I | c.C5401A | p.L1801M | nonsynonymous SNV |
| 272 | NBEAL1 | c.G3463T | p.E1155X | stopgain |
| 273 | PDE12 | c.T350C | p.V117A | nonsynonymous SNV |
| 274 | GDF5OS | c.T607C | p.F203L | nonsynonymous SNV |
| 275 | SYNE2 | c.C18595G | p.Q6199E | nonsynonymous SNV |
| 276 | PARP4 | c.A610G | p.I204V | nonsynonymous SNV |
| 277 | SNRNP200 | c.G4165A | p.V1389I | nonsynonymous SNV |
| 278 | EPPK1 | c.T15265A | p.X5089R | stoploss |
| 279 | ADCK5 | c.C51A | p.S17R | nonsynonymous SNV |
| 280 | PRDM5 | c.A491T | p.K164I | nonsynonymous SNV |
| 281 | OR2AG1 | c.G447A | p.W149X | stopgain |
| 282 | LILRB4 | c.G668A | p.G223D | nonsynonymous SNV |
| 283 | MUSK | c.A1991G | p.N664S | nonsynonymous SNV |
| 284 | GRK7 | c.G1204A | p.D402N | nonsynonymous SNV |
| 285 | HLA-DOB | c.C19G | p.P7A | nonsynonymous SNV |
| 286 | KIAA0513 | c.G37A | p.D13N | nonsynonymous SNV |
| 287 | ZNF708 | c.T961G | p.S321A | nonsynonymous SNV |
| 288 | ZDHHC1 | c.G341A | p.S114N | nonsynonymous SNV |
| 289 | FRAS1 | c.G5374A | p.A1792T | nonsynonymous SNV |
| 290 | TOR2A | c.C463T | p.R155C | nonsynonymous SNV |
| 291 | UNC13D | c.C2896T | p.R966W | nonsynonymous SNV |
| 292 | ATP5SL | c.G688A | p.E230K | nonsynonymous SNV |
| 293 | KIAA1671 | c.C613A | p.P205T | nonsynonymous SNV |
| 294 | OSBP2 | c.C947G | p.T316R | nonsynonymous SNV |
| 295 | DNAH3 | c.G1923T | p.K641N | nonsynonymous SNV |
| 296 | HTR2A | c.G55A | p.V19M | nonsynonymous SNV |
| 297 | BNIPL | c.G566A | p.R189H | nonsynonymous SNV |
| 298 | C2orf66 | c.T31G | p.F11V | nonsynonymous SNV |
| 299 | CLINT1 | c.T1704A | p.N568K | nonsynonymous SNV |
| 300 | IQCA1L | c.A1219G | p.N407D | nonsynonymous SNV |
| 301 | RASGRP1 | c.T1096A | p.Y366N | nonsynonymous SNV |
| 302 | SHPK | c.A1237G | p.M413V | nonsynonymous SNV |
| 303 | ANKHD1 | c.G3142A | p.D1048N | nonsynonymous SNV |
| 304 | NKX2-2 | c.G365C | p.G122A | nonsynonymous SNV |
| 305 | ZNF689 | c.C283T | p.P95S | nonsynonymous SNV |
| 306 | FARS2 | c.C737T | p.T246M | nonsynonymous SNV |
| 307 | ZNF92 | c.A1171G | p.T391A | nonsynonymous SNV |
| 308 | NUDT9 | c.C167T | p.A56V | nonsynonymous SNV |
| 309 | WWC2 | c.C2935T | p.R979C | nonsynonymous SNV |
| 310 | KRTAP10-4 | c.A31G | p.S11G | nonsynonymous SNV |
| 311 | TSC1 | c.A1757G | p.K586R | nonsynonymous SNV |
| 312 | CSF1 | c.C635T | p.A212V | nonsynonymous SNV |
| 313 | IQCH | c.G202C | p.V68L | nonsynonymous SNV |
| 314 | GLIS3 | c.A82G | p.I28V | nonsynonymous SNV |
| 315 | OBSCN | c.C6253T | p.H2085Y | nonsynonymous SNV |
| 316 | CNTN2 | c.C1309G | p.L437V | nonsynonymous SNV |
| 317 | ABCC5 | c.G1718A | p.R573H | nonsynonymous SNV |
| 318 | TMEM41A | c.A149G | p.E50G | nonsynonymous SNV |
| 319 | TMEM8A | c.C1688T | p.A563V | nonsynonymous SNV |
| 320 | CYTIP | c.C788T | p.T263M | nonsynonymous SNV |
| 321 | NHSL1 | c.G1426A | p.A476T | nonsynonymous SNV |
| 322 | GAS6 | c.C1739T | p.S580L | nonsynonymous SNV |
| 323 | GMPS | c.A1583C | p.Y528S | nonsynonymous SNV |
| 324 | SLC39A7 | c.T160C | p.F54L | nonsynonymous SNV |
| 325 | PP2D1 | c.G605A | p.C202Y | nonsynonymous SNV |
| 326 | OBSCN | c.G18508A | p.E6170K | nonsynonymous SNV |
| 327 | PLEKHG4B | c.G629C | p.S210T | nonsynonymous SNV |
| 328 | KIF24 | c.T3617G | p.L1206R | nonsynonymous SNV |
| 329 | OR12D2 | c.G79A | p.V27M | nonsynonymous SNV |
| 330 | CRYBG1 | c.C3055T | p.P1019S | nonsynonymous SNV |
| 331 | CSRNP3 | c.C1110G | p.D370E | nonsynonymous SNV |
| 332 | CORO1A | c.C1097A | p.P366H | nonsynonymous SNV |
| 333 | OR1F1 | c.C537G | p.C179W | nonsynonymous SNV |
| 334 | PARP15 | c.A1304G | p.H435R | nonsynonymous SNV |
| 335 | LRRC8E | c.2112delG | p.L704fs | frameshift deletion |
| 336 | SURF2 | c.G637A | p.G213S | nonsynonymous SNV |
| 337 | ALKBH2 | c.A421G | p.I141V | nonsynonymous SNV |
| 338 | RETSAT | c.G1756A | p.A586T | nonsynonymous SNV |
| 339 | DNAH7 | c.T1139G | p.M380R | nonsynonymous SNV |
| 340 | FBXW7 | c.377dupA | p.D126fs | frameshift insertion |
| 341 | LGALS3BP | c.G25A | p.V9M | nonsynonymous SNV |
| 342 | GRIK5 | c.T1490C | p.V497A | nonsynonymous SNV |
| 343 | MED15 | c.762_763insCAG | p.Q254delinsQQ | nonframeshift insertion |
| 344 | CEACAM20 | c.G1409A | p.C470Y | nonsynonymous SNV |
| 345 | FHOD3 | c.A2888G | p.K963R | nonsynonymous SNV |
| 346 | SEMA6A | c.A2960C | p.Q987P | nonsynonymous SNV |
| 347 | TBC1D23 | c.G721A | p.A241T | nonsynonymous SNV |
| 348 | FMNL3 | c.G2662A | p.A888T | nonsynonymous SNV |
| 349 | SOBP | c.C1729T | p.P577S | nonsynonymous SNV |
| 350 | KNL1 | c.C2447G | p.T816S | nonsynonymous SNV |
| 351 | TTN | c.A61906T | p.N20636Y | nonsynonymous SNV |
| 352 | WFS1 | c.G2495A | p.R832H | nonsynonymous SNV |
| 353 | GIGYF1 | c.G2989A | p.G997S | nonsynonymous SNV |
| 354 | CROCC2 | c.A4099G | p.I1367V | nonsynonymous SNV |
| 355 | CABP1 | c.C61G | p.Q21E | nonsynonymous SNV |
| 356 | S100A7L2 | c.C135G | p.N45K | nonsynonymous SNV |
| 357 | KIAA1671 | c.G1931T | p.R644L | nonsynonymous SNV |
| 358 | NR2E3 | c.G230A | p.R77Q | nonsynonymous SNV |
| 359 | PIH1D1 | c.522delC | p.I174fs | frameshift deletion |
| 360 | MRPL36 | c.A49C | p.S17R | nonsynonymous SNV |
| 361 | ADAMTS10 | c.G401C | p.S134T | nonsynonymous SNV |
| 362 | ABCF3 | c.G787T | p.D263Y | nonsynonymous SNV |
| 363 | APOA5 | c.C259A | p.Q87K | nonsynonymous SNV |
| 364 | LOC645177 | c.A593G | p.D198G | nonsynonymous SNV |
| 365 | SSPO | c.11582dupA | p.Y3861_C3862delinsX | stopgain |
| 366 | PTS | c.A11G | p.E4G | nonsynonymous SNV |
| 367 | PROP1 | c.150delA | p.G50fs | frameshift deletion |
| 368 | BEST3 | c.A1094C | p.Q365P | nonsynonymous SNV |
| 369 | AVPR1B | c.G571C | p.G191R | nonsynonymous SNV |
| 370 | PLXNB3 | c.G5218A | p.V1740M | nonsynonymous SNV |
| 371 | CCDC168 | c.T20387C | p.V6796A | nonsynonymous SNV |
| 372 | FAM110A | c.C545T | p.P182L | nonsynonymous SNV |
| 373 | NEURL2 | c.T773C | p.V258A | nonsynonymous SNV |
| 374 | KATNB1 | c.C1319G | p.P440R | nonsynonymous SNV |
| 375 | HELZ2 | c.C3889T | p.L1297F | nonsynonymous SNV |
| 376 | CYP3A7-CYP3A51P | c.G1525T | p.D509Y | nonsynonymous SNV |
| 377 | JRK | c.G455A | p.S152N | nonsynonymous SNV |
| 378 | GIPC1 | c.C677T | p.A226V | nonsynonymous SNV |
| 379 | WWP2 | c.G514C | p.A172P | nonsynonymous SNV |
| 380 | NCR1 | c.C244A | p.Q82K | nonsynonymous SNV |
| 381 | EIF2B3 | c.G517C | p.E173Q | nonsynonymous SNV |
| 382 | SKIV2L | c.G2801C | p.R934P | nonsynonymous SNV |
| 383 | NPC1L1 | c.G529A | p.V177I | nonsynonymous SNV |
| 384 | RCL1 | c.C882A | p.D294E | nonsynonymous SNV |
| 385 | IQCK | c.A1C | p.M1L | nonsynonymous SNV |
| 386 | MRPS34 | c.G432C | p.K144N | nonsynonymous SNV |
| 387 | KCNH2 | c.C2948T | p.T983I | nonsynonymous SNV |
| 388 | TRIM64C | c.G136T | p.G46C | nonsynonymous SNV |
| 389 | COL6A2 | c.G1598A | p.R533H | nonsynonymous SNV |
| 390 | NELL1 | c.C955A | p.H319N | nonsynonymous SNV |
| 391 | SUN1 | c.G2143A | p.V715I | nonsynonymous SNV |
| 392 | KMT2D | c.G3701C | p.G1234A | nonsynonymous SNV |
| 393 | IGSF3 | c.C3123G | p.D1041E | nonsynonymous SNV |
| 394 | KIAA1161 | c.C69G | p.Y23X | stopgain |
| 395 | PLXNB1 | c.C1303T | p.P435S | nonsynonymous SNV |
| 396 | PALMD | c.C343T | p.R115W | nonsynonymous SNV |
| 397 | RAB26 | c.G368A | p.R123K | nonsynonymous SNV |
| 398 | LIG3 | c.G2942A | p.S981N | nonsynonymous SNV |
| 399 | IFI44L | c.G1170T | p.M390I | nonsynonymous SNV |
| 400 | LOC645177 | c.G2190C | p.R730S | nonsynonymous SNV |
| 401 | ERN2 | c.G2753C | p.R918T | nonsynonymous SNV |
| 402 | CAMK2G | c.G1507T | p.D503Y | nonsynonymous SNV |
| 403 | OR6K2 | c.671_672del | p.A224fs | frameshift deletion |
| 404 | PLXNB3 | c.G1981A | p.G661S | nonsynonymous SNV |
| 405 | GYG2 | c.C332A | p.P111H | nonsynonymous SNV |
| 406 | MMP16 | c.C928T | p.R310C | nonsynonymous SNV |
| 407 | PPP4R1 | c.A2642G | p.Y881C | nonsynonymous SNV |
| 408 | SLC27A3 | c.C392T | p.A131V | nonsynonymous SNV |
| 409 | ADSSL1 | c.A1C | p.M1L | nonsynonymous SNV |
| 410 | LIPA | c.G490T | p.G164C | nonsynonymous SNV |
| 411 | GPR179 | c.C1498T | p.P500S | nonsynonymous SNV |
| 412 | ZSCAN5A | c.G1391A | p.G464E | nonsynonymous SNV |
| 413 | LAMA3 | c.C7406G | p.A2469G | nonsynonymous SNV |
| 414 | IGLL1 | c.G404T | p.C135F | nonsynonymous SNV |
| 415 | MSH5 | c.G1794C | p.K598N | nonsynonymous SNV |
| 416 | PCIF1 | c.A799G | p.I267V | nonsynonymous SNV |
| 417 | SETD1A | c.G2626A | p.E876K | nonsynonymous SNV |
| 418 | ISG20L2 | c.G471C | p.Q157H | nonsynonymous SNV |
| 419 | ARPP21 | c.G756T | p.L252F | nonsynonymous SNV |
| 420 | UST | c.G388A | p.G130R | nonsynonymous SNV |
| 421 | CXXC1 | c.G1820A | p.R607H | nonsynonymous SNV |
| 422 | ABCA8 | c.A1514T | p.D505V | nonsynonymous SNV |
| 423 | ZNF180 | c.T122C | p.V41A | nonsynonymous SNV |
| 424 | CNKSR1 | c.C851G | p.P284R | nonsynonymous SNV |
| 425 | ENOPH1 | c.G377A | p.R126H | nonsynonymous SNV |
| 426 | WDR60 | c.C941T | p.A314V | nonsynonymous SNV |
| 427 | OR1A2 | c.G247T | p.A83S | nonsynonymous SNV |
| 428 | IKBIP | c.626delA | p.N209fs | frameshift deletion |
| 429 | ASCC3 | c.C2563T | p.R855X | stopgain |
| 430 | CHST14 | c.T635C | p.V212A | nonsynonymous SNV |
| 431 | CUL7 | c.C4762A | p.L1588I | nonsynonymous SNV |
| 432 | SRSF7 | c.G286T | p.A96S | nonsynonymous SNV |
| 433 | EXOC4 | c.T1946G | p.L649W | nonsynonymous SNV |
| 434 | PLEKHG4B | c.A770G | p.H257R | nonsynonymous SNV |
| 435 | TMEM132A | c.C1795T | p.R599C | nonsynonymous SNV |
| 436 | OPA3 | c.A416T | p.Q139L | nonsynonymous SNV |
| 437 | SLC25A47 | c.C923T | p.T308I | nonsynonymous SNV |
| 438 | CROCC | c.G3371A | p.R1124Q | nonsynonymous SNV |
| 439 | GALNT13 | c.C840A | p.D280E | nonsynonymous SNV |
| 440 | AP5Z1 | c.C281G | p.S94C | nonsynonymous SNV |
| 441 | TNRC18 | c.C2293T | p.P765S | nonsynonymous SNV |
| 442 | ARFGAP1 | c.G1046C | p.R349P | nonsynonymous SNV |
| 443 | MYH8 | c.G3920T | p.S1307I | nonsynonymous SNV |
| 444 | CNTNAP2 | c.721dupA | p.L240fs | frameshift insertion |
| 445 | AP5Z1 | c.G333C | p.Q111H | nonsynonymous SNV |
| 446 | CLCN2 | c.G152T | p.G51V | nonsynonymous SNV |
| 447 | COL25A1 | c.C472T | p.Q158X | stopgain |
| 448 | CBL | c.1096_1112del | p.E366fs | frameshift deletion |
| 449 | SLC35G3 | c.C677A | p.S226Y | nonsynonymous SNV |
| 450 | GPLD1 | c.A1829G | p.H610R | nonsynonymous SNV |
| 451 | TBXAS1 | c.C1352A | p.T451N | nonsynonymous SNV |
| 452 | DDR2 | c.G2239A | p.A747T | nonsynonymous SNV |
| 453 | NEFM | c.C682T | p.Q228X | stopgain |
| 454 | MGEA5 | c.G137A | p.G46E | nonsynonymous SNV |
| 455 | DNMT3A | c.G890C | p.W297S | nonsynonymous SNV |
| 456 | ELMO3 | c.A2210G | p.K737R | nonsynonymous SNV |
| 457 | NCOR1 | c.C4556A | p.S1519X | stopgain |
| 458 | ATP2B3 | c.G1945A | p.A649T | nonsynonymous SNV |
| 459 | IQUB | c.G143T | p.G48V | nonsynonymous SNV |
| 460 | CLTC | c.G2806A | p.E936K | nonsynonymous SNV |
| 461 | PROM2 | c.G1870T | p.V624F | nonsynonymous SNV |
| 462 | DDX42 | c.C1649A | p.P550Q | nonsynonymous SNV |
| 463 | LDB3 | c.1298_1345del | p.433_449del | nonframeshift deletion |
| 464 | MUC6 | c.G895A | p.V299M | nonsynonymous SNV |
| 465 | TPTE | c.530delT | p.I177fs | frameshift deletion |
| 466 | CCT6B | c.G771T | p.E257D | nonsynonymous SNV |
| 467 | PRAC2 | c.102delT | p.C34fs | frameshift deletion |
| 468 | ANKRD6 | c.G274A | p.A92T | nonsynonymous SNV |
| 469 | ZNF316 | c.T1378C | p.S460P | nonsynonymous SNV |
| 470 | LAT2 | c.G94T | p.G32C | nonsynonymous SNV |
| 471 | OTOP1 | c.C310A | p.L104M | nonsynonymous SNV |
| 472 | TDRD10 | c.C748T | p.R250C | nonsynonymous SNV |
| 473 | PI4KB | c.A524G | p.Y175C | nonsynonymous SNV |
| 474 | PNPLA6 | c.1118dupT | p.M373fs | frameshift insertion |
| 475 | HMCN2 | c.C9602T | p.T3201I | nonsynonymous SNV |
| 476 | SSPO | c.G13733A | p.R4578H | nonsynonymous SNV |
| 477 | CGNL1 | c.G3085A | p.D1029N | nonsynonymous SNV |
| 478 | RNASEH1 | c.C395T | p.T132M | nonsynonymous SNV |
| 479 | RAPGEF3 | c.C1214T | p.A405V | nonsynonymous SNV |
| 480 | NFATC4 | c.486_487insGGGGGTGCT | p.G162delinsGGGA | nonframeshift insertion |
| 481 | ZNF395 | c.G1286A | p.S429N | nonsynonymous SNV |
| 482 | SARDH | c.G2452A | p.A818T | nonsynonymous SNV |
| 483 | LRRTM1 | c.G923T | p.S308I | nonsynonymous SNV |
| 484 | CCDC150 | c.840delA | p.Q280fs | frameshift deletion |
| 485 | OR14A16 | c.C357G | p.D119E | nonsynonymous SNV |
| 486 | MNS1 | c.604delA | p.K202fs | frameshift deletion |
| 487 | ZSCAN18 | c.C425T | p.A142V | nonsynonymous SNV |
| 488 | C1orf87 | c.C864A | p.S288R | nonsynonymous SNV |
| 489 | POLR2A | c.G2362A | p.V788I | nonsynonymous SNV |
| 490 | FGFR2 | c.2287_2288del | p.T763fs | frameshift deletion |
| 491 | PTPN11 | c.G181T | p.D61Y | nonsynonymous SNV |
| 492 | STAC2 | c.C384G | p.H128Q | nonsynonymous SNV |
| 493 | SEPT5 | c.685_687del | p.229_229del | nonframeshift deletion |
| 494 | RNF17 | c.T2438A | p.F813Y | nonsynonymous SNV |
| 495 | RTEL1 | c.974_991del | p.325_331del | nonframeshift deletion |
| 496 | SPTA1 | c.C178T | p.R60X | stopgain |
| 497 | PTPN6 | c.A1202G | p.D401G | nonsynonymous SNV |
| 498 | NRG1 | c.C1864T | p.R622C | nonsynonymous SNV |
| 499 | RAB11FIP3 | c.C424G | p.P142A | nonsynonymous SNV |
| 500 | PIK3CA | c.C1636A | p.Q546K | nonsynonymous SNV |
| 501 | ABL1 | c.C8A | p.E3A | nonsynonymous SNV |
| 502 | ADCK5 | c.1256_1257insGGGGGTGCAAGGTGA | p.L419delinsLGVQGE | nonframeshift insertion |
